# Beyond Static Tethering at Membrane Contact Sites: Structural Dynamics and Functional Implications of VAP Proteins

**DOI:** 10.3390/molecules30061220

**Published:** 2025-03-08

**Authors:** Takashi S. Kodama, Kyoko Furuita, Chojiro Kojima

**Affiliations:** 1 Institute for Protein Research, Osaka University, 3-2 Yamadaoka, Suita, Osaka 565-0871, Japan; k-furuit@protein.osaka-u.ac.jp; 2 Graduate School of Engineering Science, Yokohama National University, Tokiwadai 79-5, Hodogaya-ku, Yokohama 240-8501, Japan

**Keywords:** VAP, MSP, coiled coil, transmembrane, IDR, MCS, WGD

## Abstract

The membranes surrounding the eukaryotic cell and its organelles are continuously invaginating, budding, and undergoing membrane fusion–fission events, which enable them to perform functions not found in prokaryotic cells. In addition, organelles come into close contact with each other at membrane contact sites (MCSs), which involve many types of proteins, and which regulate the signaling and transport of various molecules. Vesicle-associated membrane protein (VAMP)-associated protein (VAP) is an important factor involved in the tethering and contact of various organelles at MCSs in almost all eukaryotes and has attracted attention for its association with various diseases, mainly neurodegenerative diseases such as amyotrophic lateral sclerosis (ALS). However, the detailed mechanism of its functional expression remains unclear. In this review, we quantitatively discuss the structural dynamics of the entire molecule, including intrinsically disordered regions and intramolecular and intermolecular interactions, focusing on the vertebrate VAP paralogs VAPA and VAPB. Molecular phylogenetic and biophysical considerations are the basis of the work.

## 1. Introduction

One of the major characteristics of eukaryotic cells is their abundance of organelles, which efficiently share functions and work in concert. These organelles are surrounded by membranes of defined protein and lipid composition. In eukaryotic cells, the constant invagination, budding, fusion, and fission of the plasma membrane or the membranes surrounding each intracellular organelle play a major role in achieving functions that are not found in prokaryotic cells.

Concerning the functional connections between these organelles, it has been established in the last decade that, besides membrane fusion [1] and vesicle trafficking, close contact between organelles not followed by membrane fusion—called membrane contact sites (MCSs)—plays an important role in signaling and material exchange between intracellular compartments. Many types of proteins are involved in the function of MCSs. (For MCSs between the ER and other intracellular organelles, see [2,3,4,5]; for a more comprehensive review of MCS, see [6]). VAMP-associated proteins (VAPs) are important factors that play a role in the mutual anchoring and contact between the ER and various intracellular organelles in almost all eukaryotes [7,8,9,10,11,12,13,14]. However, the detailed mechanism of its functional expression remains unclear.

In 1995, scs2 was cloned as a factor that suppresses the inositol requirement of CSE1 and hac1/ire15 mutants of budding yeast (*Saccharomyces cerevisiae*) [15]. In the same year, a 33-kD protein that interacts with vesicle-associated membrane protein (VAMP, or synaptobrevin) was cloned from the marine mollusk *Aplysia californica* and named VAMP-associated protein 33 (VAP-33), suggesting that it may play a role in synaptic vesicles [16]. In 1998, scs2 and VAP-33 were thought to be homologs [17]. In 1998, a homolog of Aplysia VAP-33 was also found in the humans [18], and it was thought that it might exist in all eukaryotes.

In 1999, VAPA, identified as hVAP-33, and its paralogous gene VAPB were identified in humans and rats [19]. The existence of VAPC, a splicing variant of VAPB, has also been demonstrated, but its expression in vivo has not been confirmed. Subsequent studies have revealed that VAPA/B interacts with different proteins at various sites in the cell and is involved in various functions. For comprehensive reviews of each topic, see, for example, the following reviews and references. These include tethering for the coordination of organelles and the endoplasmic reticulum [8,12,14,20], lipid trafficking [21,22], autophagy [23], ion homeostasis [24,25], viral entry pathways [12], and the unfolded protein response [26,27]. Furthermore, since its relationship with amyotrophic lateral sclerosis (ALS) was reported [28,29], it has also attracted attention for its association with various diseases, mainly neurodegenerative diseases, as discussed in [8,12].

Among these diverse interactions and functions, a highly conserved sequence at the N-terminus of VAP that may be functionally important has been recognized since the initial series of cloning [16,18,19] and has come to be called the major sperm protein domain (MSPd). It has been widely studied since its interactions with the “two phenylalanines in an acidic tract” (FFAT) motif of other molecules [30] were revealed. There are many previous studies and reviews on the interaction between MSPd and FFAT of other proteins in terms of function and, especially, in relation to diseases, so please refer to them for details [8,31,32,33].

Based on the sequence characteristics, VAP is known to have a coiled-coil domain (CCd) and a transmembrane domain (TMd), in addition to MSPd [18,19]. An in vitro binding assay showed TMd is important for the homo- and hetero-oligomerization of human VAPA and VAPB [19]. Experiments using epitope-tagged constructs in both cell-free and cellular systems have directly demonstrated that the TMd of human VAPB has a transmembrane topology [34].

VAPs are normally located on the ER membrane, with their N-termini in the cytoplasm. However, when present at the plasma membrane, this topology is reversed, with the N-terminus exposed on the extracellular side of the plasma membrane. The conversion of the VAP topology appears to occur somewhere along the classical Golgi-dependent secretory pathway [35].

In this review, we provide an overview of the properties of VAP that have been elucidated thus far and verify some points. We then perform a molecular phylogenetic analysis of each region of VAP from the levels of primary, secondary, tertiary, and quaternary structure and combine these to draw a quantitative picture of how the structural and dynamic properties of the entire molecule are likely to be related to function.

## 2. Origin of VAP Homologs and Paralogs

### 2.1. Origin of VAP Homologs

**Figure 1:**
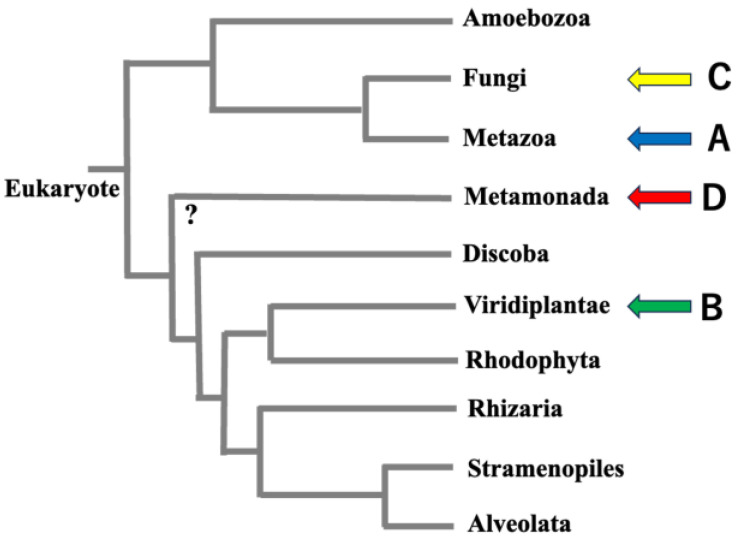
Evolutionary tree of eukaryotes. Vertebrates belong to the branch indicated by the blue arrow (**A**), and land plants belong to the branch indicated by the green arrow (**B**). It is noteworthy that both have MSPd, CCd, and TMd, indicating that the domain architecture was already present in the deepest branches of these eukaryotes. Some yeasts belonging to the Ascomycota phylum in the branch indicated by the yellow arrow (**C**) lack CCd. However, this phylogenetic tree shows that this is not because they have not yet acquired CCd during evolution, but because they have lost the CCd that they originally had. Conversely, some, but not all, of the protists in the branch indicated by the red arrow (**D**) do not have VAP genes. The position of Metamonada in the eukaryotic evolutionary tree, indicated by a question mark, remains a difficult problem [37,38]. Please note that this figure is intended to show the branching relationships diagrammatically.

It is almost certain that VAP homologs exist in all eukaryotes because they have been shown to exist in mammals, plants, and fungi, which belong to the lineages considered to be the most deeply branched among eukaryotes (Figure 1 and Figure S1). However, among protists, there are some species, such as *Trichomonas vaginalis* G3 (NCBI:taxid412133) and *Giardia intestinalis* (NCBI:taxid5741), for which VAP homologs cannot be confirmed by BLASTp searches [36] against databases that have quality ratings in the NCBI Genome at the chromosome or complete assembly level (Figure 1). When examining the phylogenetic relationships of species for which VAP homologs could not be confirmed, VAP homologs were assigned to the ends of several branches. Thus, these organisms cannot be considered monophyletic. This suggests that we are not observing a process in which the common ancestor of eukaryotes did not have the VAP homologous gene, and a later lineage evolved to acquire the VAP homologous gene. Rather, the most ancient common ancestor of eukaryotes already had the VAP homologous gene, and the VAP gene has degenerated in a small number of species during subsequent divergence.

VAP is known to play an important role in tethering between the ER and mitochondria. For example, VAPB on the ER has been reported to tether several target molecules such as PTPIP51 on the mitochondrial outer membrane [39,40,41,42,43], VPS13 A and D [44,45], and MIGA-2 [46,47]. The presence of mitochondrial-associated genes and organelles in mitochondria-deficient protists may indicate that these protists have secondarily lost typical mitochondria separately in different lineages [48,49,50,51,52,53]. This supports the idea that the VAP genes acquired in ancestral eukaryotes were lost secondarily during evolution in anaerobic environments. These facts suggest a link between VAP genes and the (loss of) mitochondria during the evolution of anaerobic protists.

### 2.2. Origin and Significance of VAP Paralogs VAPA and VAPB

The orthologs of two paralogous genes, VAPA and VAPB, exist in vertebrates [8]. We confirmed the presence of two paralogous genes (VAPA and VAPB) in many branches of the vertebrate phylogenetic tree. However, two paralogous genes could not be confirmed in the cyclostomes, the lineage that branches off at the deepest part of the vertebrate phylogenetic relationship (lampreys and hagfish). Therefore, it seems more accurate to state that the two independent genes, VAPA and VAPB, are not present in all vertebrates, but rather in all gnathostomes within vertebrates (see the MSA diagram of Eukaryotes and Vertebrates).

The biological significance of the fact that only vertebrates have VAPA and VAPB paralogs, and that their properties are highly conserved, is related to properties found only in vertebrates (autapomorphy). Among the properties shared by vertebrates (including vertebrates in the strict sense of the word and hagfish) listed by Nielsen et al. in 1995 [54], the existence of the neural crest seems to be important, especially when considered together with the high expression levels of both VAPA/B in the brain [55,56]. The neural crest is associated with ganglions, peripheral motor ganglions, sensory neurons, and higher motor neurons. Therefore, the existence of VAPA/B paralogs may play an important role in the developmental stages of these structures and their subsequent maintenance.

*Saccharomyces cerevisiae* and *Schizosaccharomyces pombe* have two VAP homologs, scs2 and scs22, on different chromosomes. Thus, organisms with multiple VAP genes are also found in nonvertebrate tissues, but by comparing the sequence characteristics, which will be explained later, we conclude that these are not direct orthologs of vertebrate VAPA and VAPB but formed independently.

## 3. Domain Architecture and Domain Boundaries of VAPA and VAPB

### 3.1. Domain Architecture

**Figure 2:**
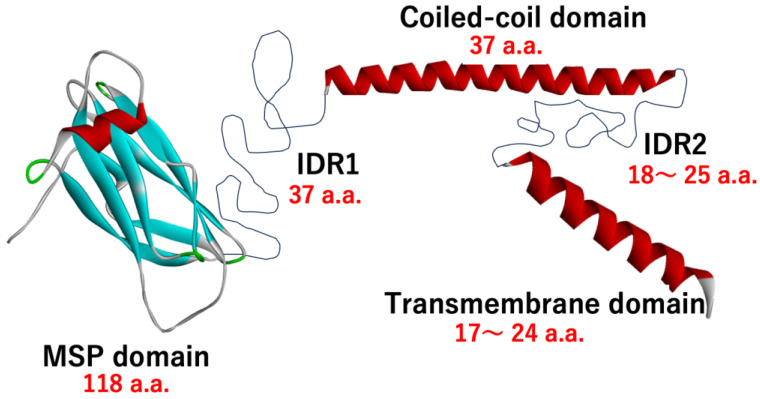
Schematic description of the domain architecture of VAPs. The structure of the MSP domain is an experimentally determined NMR structure of human VAPA (PDB ID:2rr3). Other domains are a schematic representation of human VAPA.

As predicted by earlier studies [18,19], VAPA and VAPB share a common domain architecture, consisting of a major sperm protein (MSP) domain, a coiled-coil domain, a transmembrane domain, and a region connecting them. In this paper, we refer to these parts of the architecture as the MSP domain (MSPd), Intrinsically Disordered Region 1 (IDR1), Coiled-coil domain (CCd), Intrinsically Disordered Region 2 (IDR2), and Transmembrane domain (TMd), respectively, from the N-terminus (Figure 2).

### 3.2. Domain Boundaries of Regions MSPd, IDR1, CCd, IDR2, and TMd

In human sequences, the MSP domain is residues 14–131 in human VAPA (corresponding to 7–124 in human VAPB), based on a sequence analysis using Prosite in Uniprot (ProRule PRU00132). Hereafter, the residue numbers are the numbers in the sequences of human VAPA (Uniprot canonical sequence), unless otherwise specified. Since residues 14–131 correspond to the rigid part in the NMR structure (PDB ID:2rr3), it is believed that the structural domain boundary is at these positions.

The existence of CCd and TMd is mainly based on a sequence analysis using bioinformatics tools; the structure and domain boundaries have not been confirmed experimentally. In Uniprot, the CCd is 169–205 in human VAPA (corresponding to 163–200 in human VAPB) as a result of sequence analysis, and NPS@ coiled-coil prediction also predicts that 169–205 in human VAPA is the CCd with a probability of over 99%. In addition, the structure predicted by AlphaFold3 [57] predicts that 167–207 is a helix structure. The domain boundaries of the CCd are consistent.

Regarding the TMd, the analysis by SOSUI, which was used for the prediction by Nishimura et al. in 1999 [19], predicted that the TMd is 226–248 in human VAPA. Although these regions are generally predicted to be TMds by other prediction methods, the predicted positions of the N-terminus side differ significantly depending on the prediction program, with the difference being up to two turns of the helix (TMHMM 2.0 [58]: 231–247 in human VAPA, DeepTMHMM [59]: 231–247 in human VAPA, and TOPCONS [60]: 227–247 (224–247 and 225–247 are also possible) in human VAPA).

From the lipid bilayer perspective, the length of the membrane-embedded helix of a TMd is closely related to the tilt angle of the helix relative to the normal angle of the membrane. However, the inclination angles of the transmembrane helices vary and may fluctuate [61]. Thus, strictly defining a single boundary may be an illusory goal. In this review, we considered the boundaries of the TMd to be in the range of 224–247 to 231–247 in human VAPA (corresponding to 219–241 to 226–241 in human VAPB).

**Figure 3:**
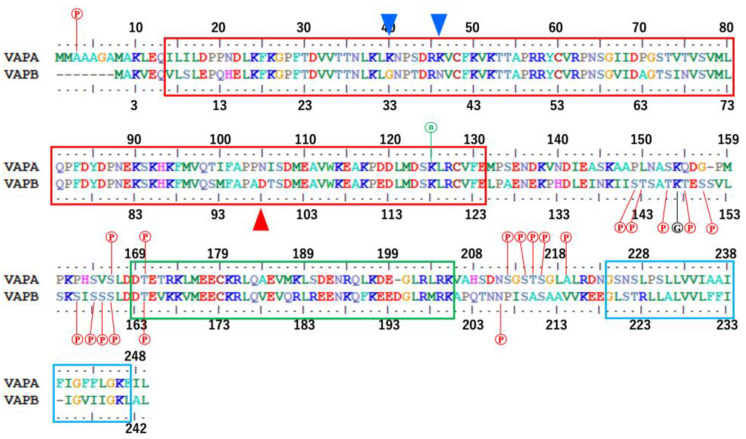
Consensus sequences of vertebrate VAPA and VAPB. The consensus sequences were generated on the ConSurf server [63] using the MSA in Figure S2. The phosphorylation sites are described in the PTM (Post-Translational Modification) section of the Uniprot (Release 2024_06) entry: Q9P0L0 (human VAPA) and entry: O95292 (human VAPB) are indicated by red Ⓟ, the positions of the N6-acetyllysine are indicated by green ⓝ, and the glycyl-lysine isopeptide by black Ⓖ. The area enclosed by the red box represents the MSPd, the green box represents the CCd, and the light-blue box represents the TMd. The blue triangles indicate the positions of two basic residues that are characteristically conserved in VAPA, and the red triangle indicates the position of one acidic residue that is characteristically conserved in VAPB. Note that VAPA residue 3 and 219 and VAPB residue 206 are serine residues in the human sequences, corresponding to alanine and asparagine residues in the consensus sequence, respectively.

The regions between the MSP domain and the CCd, and between the CCd and the TM domain, have not been recognized as specific domains from a sequence analysis. When this region is analyzed using disorder region prediction programs, such as PSI-Pred and SACRATCH, it is predicted to be an intrinsically disordered region (IDR). In this review, the region between MSPd and CCd is referred to as IDR1, and the region between CCd and TMd is referred to as IDR2. If the boundaries of MSPd, CCd, and TMd are defined as above, the length of IDR1 is 37 a.a. in human VAPA (38 a.a. in human VAPB), and the length of IDR2 is 18–25 a.a. in both VAPA and VAPB (Figure 2 and Figure 3). These structures and exact boundaries have not been experimentally confirmed, but this part is also predicted as an IDR with a clearly low pLDDT in the structure predicted by AlphaFold3. The IDR predicted by AlphaFold3 with a low pLDDT is not a conditional IDR but is considered to have a high probability of being a region with a random structure [62], so this part is thought to be an IDR that continues to change shape over a fairly wide conformational space while bending.

## 4. Molecular Phylogenetic Considerations

### 4.1. Molecular Phylogenetic Characteristics of the MSP Domain

MSPd was originally identified as a highly conserved region in the N-terminus of VAP homologs and was identified as a region similar to that of nematode MSP1A [17]. Figures S1–S6 show the degree of amino acid conservation in MSPd within eukaryotes and vertebrates. The degree of sequence conservation in this part is high, suggesting that the function of MSPd is evolutionarily conserved to a large extent. In addition, if we consider the degree of amino acid conservation of MSPd only for the orthologs of VAPA and VAPB, it is found that the degree of amino acid conservation is even higher. The three-dimensional structures of MSPd have been determined experimentally (Table 1).

Based on the NMR structure (PDB ID:2rr3), the degree of exposure of the side chain of each amino acid residue to the solvent was quantified using the modified STRIDE program [64], and the correspondence with the degree of evolutionary conservation was shown (Figure S7). It is well known that the degree of conservation is high for densely packed amino acid residues, which form the core of the three-dimensional structure of a protein, and for amino acids that are on the surface but interact with other molecules or are used as the enzyme active center of an enzyme. In fact, Figure S7 shows that residues with a high degree of buried amino acid side chains have a high degree of evolutionary conservation. Therefore, mapping the evolutionary conservation of amino acids onto the three-dimensional structure will provide an explanation for the evolutionary conservation of each residue.

**Figure 4:**
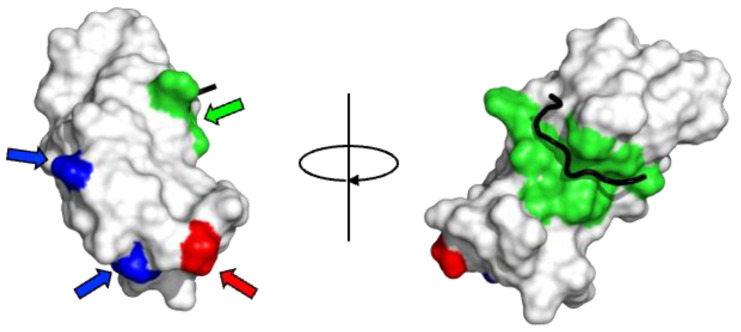
The structure of the VAP-A: OSBP (oxysterol-binding protein) FFAT complex (PDB ID:2rr3). VAPA (14–129) (surface model) and OSBP (357–367) (thick black line) are shown. Residues characteristically conserved in VAPA and VAPB are in blue and red, respectively. (Residues marked with blue and red triangles in Figure S2 are blue and red, respectively.) Residues interacting with the FFAT motif in [65] are shown in green.

It is noteworthy that the degree of evolutionary conservation is also quite high for residues with exposed amino acid side chains. The degree of conservation of physicochemical properties such as hydrophobicity, charge, and aromaticity of amino acids can also be seen in Figures S1–S6. As can be seen, when the conservation of the physicochemical properties of amino acids is also taken into consideration, the degree of conservation is quite high not only for residues present in the hydrophobic core but also for residues present on the surface. Of these, the surface residues involved in binding to the FFAT motif are almost 100% evolutionarily conserved and are likely to retain their binding properties. The residues involved in binding to the FFAT motif are mapped onto the 3D structure and shown in Figure 4.

Many other residues are present on the surface and are highly conserved. This strongly indicates that there may be an unknown essential factor for binding to the FFAT motif, or that there may be an unknown interaction with other unknown targets related to the function of VAP.

### 4.2. Molecular Phylogenetic Characteristics in Differences Between VAPA and VAPB

Another noteworthy point is the relationship between VAPA and VAPB. As is clear from the MSA results, the amino acid conservation between the two is quite high from the residues that form the core of the structure to the surface residues, and the differences between the two are very small. However, several residues on the surface differ between the two paralogs, and these clearly characterize VAPA and VAPB.

**Figure 5:**
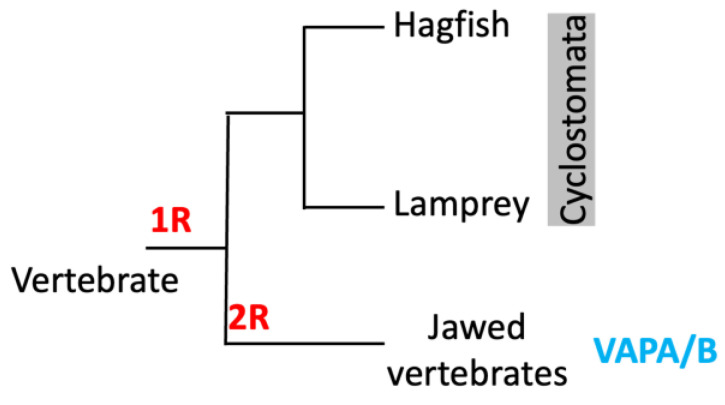
Vertebrate phylogeny and WGD. WGD events correspond to the recently proposed alternative vertebrate 2R hypothesis. The cyclostome lineage diverged from the gnathostome (jawed vertebrates) after the 1R event but before the 2R event. Therefore, the gnathostome and cyclostome lineages share the 1R but not the 2R. The presence of VAPA and VAPB orthologues in gnathostomes but not in cyclostomes suggests that VAPA/B emerged from this 2R (allotetraploidization) event.

Although more than 400 million years have passed since the evolutionary divergence of vertebrates, these features are highly conserved throughout vertebrate gnathostomes in the orthologs of VAPA and VAPB. When these features are compared with the sequences of cyclostomes among vertebrates and VAP homologs of chordates that are close to the evolutionary divergence of vertebrates, both features appear to appear simultaneously in the sequences that correspond to the ancestors of VAPA/B. Therefore, it is not possible to see which VAPA/B is closer to its evolutionary prototype. In humans, VAPA is located on chromosome 18 and VAPB on chromosome 20, but the arrangement of the genes before and after the VAP gene in each chromosome is similar, suggesting that these paralogs were generated as a result of whole-genome duplication (WGD) [66] rather than gene-level duplication. The standard 2R hypothesis [67,68,69] suggests that two WGDs (1R and 2R) occurred during early vertebrate evolution. Recently, an analysis of vertebrate genomes, including hagfish, revealed that the cyclostome lineage diverged from the gnathostome after the 1R event but before the 2R event [70]. This means that the gnathostome and cyclostome lineages share the 1R but not the 2R. The fact that VAPA and VAPB orthologues are found in gnathostomes but not in the cyclostome lineage implies that VAPA/B emerged from this 2R event. Moreover, it has been suggested that 1R was probably an autoploid event, whereas 2R was an allotetraploidization event [71,72,73]. Therefore, VAPA and VAPB must arise through WGD between heterogeneous lineages that inherited slightly different properties from a common ancestor (Figure 5).

This is interesting because the current amino acid sequence features of VAPA and VAPB appear to have been inherited by dividing and sharing some features of the original ancestor of VAP.

The abovementioned amino acid differences, which are highly conserved throughout evolution and clearly characterize the MSPd of VAPA and VAPB, are naturally assumed to be related to their functions. However, there is no reason to assume that this difference should lead to different binding abilities and selectivities of the MSPds of VAPA and VAPB for FFAT. All of the above residues are far from the interaction sites with the FFAT motif in the three-dimensional structure (see Figure 4). Therefore, these differences are unlikely to directly affect the binding abilities and selectivities for FFAT. Even if allostery is considered, its effect would be limited because it would occur at a distant site and would be mainly due to the difference in charge. The FFAT motif has various sequences, and the interaction between VAP and the FFAT motif has low sequence specificity. Therefore, it is unlikely that specificity can be precisely controlled by limited amino acid differences distal to the interaction site. Thus, the functional significance of the evolutionarily conserved differences between VAPA and VAPB must be interpreted from a perspective other than the effect on the direct interaction with FFAT.

*Saccharomyces cerevisiae* and *Schizosaccharomyces pombe* have two VAP homologous genes, scs2 and scs22, on different chromosomes. However, in nonvertebrate tissues, there is no consistent similarity between the sequence characteristics of these genes and those of VAPA and VAPB, which are common to vertebrates. Therefore, the paralogs in nonvertebrate VAPs should be considered independent entities rather than direct orthologs of vertebrate VAPA and VAPB.

### 4.3. Dimerization of MSPd

Another issue that should be discussed regarding MSPd is its dimerization. *C. elegans* MSP, a separate protein with homology to the VAP MSP domain, spontaneously dimerizes [74], raising unresolved questions about its potential multimerization and associated functions [7]. MSPd, expressed and purified as a recombinant protein, exists as a monomer in solution, and when subjected to crystal structure analysis, it has been reported to exist as a homodimer alone or as an associated form mediated by FFAT [31,75]. However, in NMR measurements in the solution, the self-association of MSPd was not observed [33,65]. In another study, MSPd seems to contribute little to the formation of the complex [19]. This is believed to be mainly due to the fact that the concentration of MSPd in the crystallization conditions is much higher than that in the NMR measurements and biochemical experiments [75], suggesting that this dimerization is a rare structure observed only under special conditions.

Examining the crystal structures listed in Table 1, the dimerization or oligomerization interfaces appear to be multiple. However, the amino acids that form the dimerization interface in these crystal structures were highly conserved in vertebrates throughout evolution. Some of these interfaces (PDB ID:3ikk) partially overlap with the FFAT motif-binding site of MSPd in the complex structure (PDB ID:2rr3), suggesting that dimerization is an inhibitory factor for interaction with the FFAT motif. This makes it difficult to assess the selective pressure behind this high degree of evolutionary conservation. For example, the conservation of the interaction surface is due to interaction with the FFAT motif, homo/hetero dimerization, or both. In the structure (PDB ID:1z9o) of MSPd in complex with FFAT, the peptide chains containing the FFAT motif are fitted into the dimer interface, while the formation of the MSPd dimer and the interaction between MSPd and the FFAT motif does not appear to be mutually exclusive.

The consideration of highly conserved amino acids reveals other interesting features. In addition to the high conservation of basic amino acids that correspond to electrostatic interactions with the acidic tract of the FFAT motif, the surrounding residues are conserved over a wide range, suggesting that MSPd has the property of gently receiving the FFAT motif over a wide surface. This is in agreement with the fact that the NMR study of the interaction between VAPA MSPd and OSBP FFAT motif showed that the interaction is not based on a highly selective and rigid single conformation, but rather on a semi-selective and multi-conformational interaction [33,65]. However, the residues in the more distant regions, which do not seem to be involved in the interaction with the FFAT motif, are also highly conserved, which suggests that there is another factor at play.

### 4.4. Differences in Structural and Dimerization Characteristics Between VAPA and VAPB

**Figure 6:**
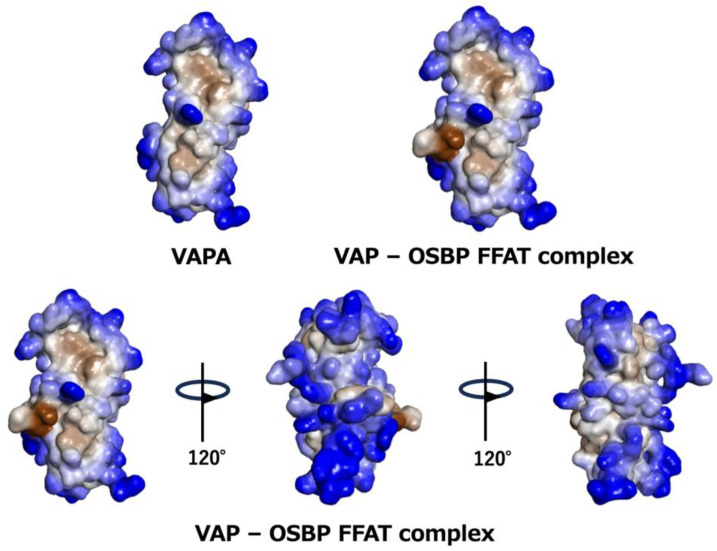
Hydrophobicity and hydrophilicity of VAPA-OSBP FFAT motif complex structure. Hydrophilic residues are in blue, and the hydrophobic surface is represented by gray and brown. (**Top**) VAPA MSPd and VAPA-OSBP FFAT motif complex. (**Bottom**) Rotated view of the whole structure of VAPA-OSBP FFAT motif complex.

Figure 6 shows hydrophilicity and hydrophobicity mapped on the NMR structure of the complex between VAPA MSPd and the OSBP FFAT motif. Most surface residues are hydrophilic (in blue in Figure 6). However, there is a hydrophobic surface near the binding site of the FFAT motif. In the complex of VAP with a peptide containing the FFAT motif of the OSBP (PDB ID:2rr3), the peptide has hydrophobic residues on the C-terminal side of the FFAT motif, resulting in a larger hydrophobic surface upon binding, whereas in other proteins, the residues on the C-terminal side of the FFAT motif are acidic, hydrophobic, or hydrophilic [76]. These differences may contribute to the ability of the FFAT motif to bind to the MSPd.

A dimerization mode in the apo state (PDB ID:3ikk) probably acts as a weak inhibitory force against the interaction between MSPd and the FFAT motif. The positive and negative charges characteristic of VAPA and VAPB are highly conserved on the surface opposite to the interface for dimer formation. If an interaction occurs between them in the heterodimer, it should suppress the association of the hydrophobic surfaces in the apo state; as a result, the ratio of the open form capable of association with the FFAT motif should increase. When the FFAT motif of OSBP binds to VAP, it may increase the interaction between the two MSPds and allow VAP to wrap around the FFAT motif. Due to the high degree of conservation of the surface amino acids, there may be weak multiple interaction sites. When these are attracted to the vicinity of the membrane by the movement of the whole molecule, as described below, the MSPd of VAP reaches a juxtamembrane region where the apparent dielectric constant of water molecules is low due to the accumulation of strong negative charges on the membrane surface. In this region, the hydrophobic interactions weaken and the hydrophobic surface of MSPd can interact with the membrane, causing the MSPd dimer to adopt the open form from which the FFAT motif can be released.

In this case, inhibition of the interaction with the FFAT motif by the MSPd dimer formation is switched to the direction of release when the VAPA and VAPB heterodimer is formed, compared with the VAPA homodimer and VAPB homodimer. Alternatively, these characteristic residues of VAPA and VAPB may provide new interaction surfaces for clustering through heterodimer–heterodimer interactions of MSPd. These considerations may provide a basis for understanding why two VAP paralogs exist in vertebrates. The VAPA and VAPB paralogs are not explained by the characteristics of the VAPA itself or VAPB itself, but it may be that the activity can be precisely increased or decreased by adjusting the ratio and amount of VAPA and VAPB expressed. VAPA/B is widely expressed in tissues throughout the body and is significantly more expressed in the brain [55,56]. In many tissues, the expression of VAPA is several times higher than that of VAPB, but the expression of VAPA and VAPB are both high and maintained almost equally in the brain. This appears to correspond to a state in which the probability of formation of the VAPA and VAPB heterodimer is high and activity is maximized. This may be a potentially important issue considering the association between altered VAP function and many neurological disorders. Many existing studies have tended to focus on either VAPA or VAPB to obtain a finer picture and characterize only a single domain. In addition, the property associated with the weak dimerization ability is expected to be a subtle property that appears only in the presence of other domains, such as CCd and TMd, as described below, and, therefore, may be an aspect that is easily overlooked in conventional studies. This is a matter that requires future research.

### 4.5. Phylogenetic Characteristics of IDR1

Although the degree of conservation of amino acids in this region may be somewhat low to define it as a clear domain, it is too distinctive to be simply considered a region of low complexity. The charged, hydrophilic, and hydrophobic groups exhibit clear periodicity (Figure 3 and Figures S1–S6). In some cases, this region may induce secondary structures or may have properties such as LLPS formation due to more ambiguous intermolecular interactions observed in the periodic repetition of opposite charges [77].

Furthermore, many phosphorylation sites are predicted in this region for both VAPA and VAPB (Figure 3), particularly for VAPB. The absolute positions of these phosphorylation sites in the primary structure are not necessarily conserved, but amino acid residues that may be phosphorylated often appear in the same areas. Because phosphorylation often occurs semi-selectively at Ser or Thr in the IDR region and the absolute position in the sequence is generally not very important, this property is conserved among many organisms. In addition, because this phosphorylation is thought to further emphasize the periodic repetition pattern of opposite charges, as described above, it may be effective in controlling the formation of an LLPS-like state. Because MSPd has a positively charged patch on the surface that interacts with the acidic tract of the FFAT motif, IDR1 may interact with the basic patch when it is acidified by phosphorylation and inhibits FFAT binding intramolecularly or intermolecularly.

The phosphorylation sites are highly localized in the C-terminal region of IDR1, especially for VAPB. This implies that this region may fold back spatially to interact with the FFAT motif-binding surface and play an autoinhibitory role. Such autoinhibition by IDR has been reported for the Wiskott–Aldrich syndrome protein (WASP) [78]. These characteristics in IDR1 may affect the average conformation of IDR1 and the interactions with other molecules.

### 4.6. Phylogenetic Characteristics of the Coiled-coil Domain

At present, there is no clear experimental confirmation of the structure of CCd in VAP, so its evaluation is mainly based on computer science. Since the 20th century, various machine-learning-based methods have been developed and used to predict the presence and association state of general CCds. However, it is still difficult to predict all types of coiled-coil structures in the protein data bank while avoiding false positives and false negatives [79]. Therefore, because it is difficult to reliably determine whether a CCd exists in an individual protein using a single method, it is desirable to make a comprehensive judgment using multiple methods and perspectives. We analyzed the presence of CCd in human VAPA and VAPB sequences using Waggawagga [80], a web-based tool for the comparative visualization of coiled-coil predictions, including four coiled-coil tools, Marcoil [81], Multicoil2 [82], Ncoils [83], and Paircoil2 [84], and three oligomerization evaluation methods (Scorer 2.0 [85], PrOCoil [86], and LOGICOIL [87]) with a window length of 21. All the tools predicted the presence of CCd in the same region, except that Multicoil2 did not predict CCd in VAPB. In addition, no CCd was predicted for Scs2p, the homolog of *S. cerevisiae*. Furthermore, no tendency to form a helix was predicted for the corresponding region of Scs2p through secondary structure prediction methods SCRATCH [88] and PSI PRED [89]. The structures of Scs2p predicted by AlphaFold3 also had no CCd (Figure S8). AlphaFold3, SCRATCH, and PSI PRED predicted that the corresponding region of VAPA/B has a high tendency to form a helix.

**Figure 7:**
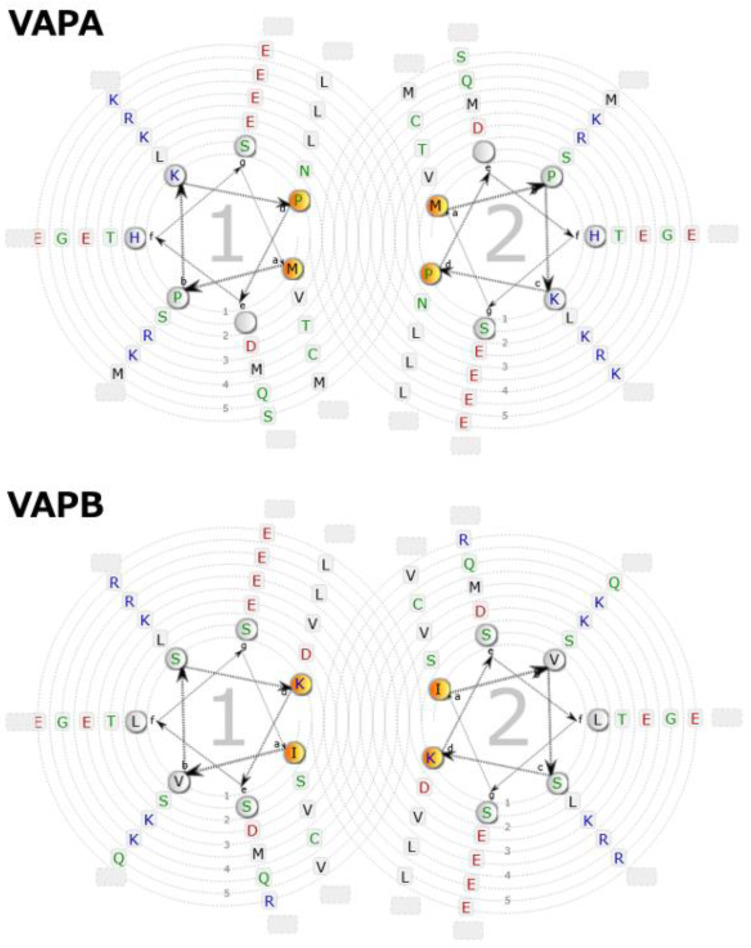
Edmundson wheels of the predicted CCd of human VAPA and VAPB. The molecules can associate through the heptad repeat interface, and the amphipathic helix is stabilized by electrostatic interactions.

We can conclude that, in human VAPA and VAPB, as previously thought, there is a fairly high probability that CCd exists in the relevant region. Because CCd is generally known as a domain that forms dimers, trimers, tetramers, or even higher aggregates [87], it is not difficult to imagine that this CCd also functions in VAP for such purposes. This is reasonable, since the CCd predicted in VAPA/B is recognized to be similar in length and characteristics to the well-known CCd in SNAREs [19]. When an Edmundson wheel is drawn for the sequence of the predicted CCd portion of VAPA/B, a typical heptad repeat is present, as shown in Figure 7, and a hydrophobic surface for intermolecular interactions is likely to form at positions a and d. In addition, strong and regular charges are arranged at positions b, c, e, f, and g, especially at positions c and g on the opposite side of the hydrophobic surface. The total length is predicted to be over 30 residues, so all these characteristics meet the general conditions for a coiled-coil heptad repeat. Furthermore, as shown by the MSA, these properties were completely conserved in vertebrates for both VAPA and VAPB (Figure 3 and Figures S2–S6).

Homology modeling can be performed for VAPB under good conditions using the dimer, trimer, or tetramer of CCd of GCN4, which is known as a typical coiled-coil region, as a template (Figure S9). Coiled-coil structures generally have various topologies, including parallel and antiparallel, with various helical contents ranging from 2 to 7 (2 to 7). They will be homodimers, heterodimers, or oligomers. It is necessary to assume multiple forms for the CCd of VAP in terms of the number of associations, association partners, and association strength, depending on the situation.

Another interesting point emerging from molecular phylogenetic studies of CCd is that certain yeasts, such as *Kluyveromyces lactis* (NCBI:taxid28985), *Saccharomyces cerevisiae* (NCBI:taxid4932), and *Schizosaccharomyces pombe* (NCBI:taxid4896), lack CCd and instead possess the longer IDR. On the other hand, all eukaryotes (except some protists, as mentioned above) have VAP homologs, and many of them are predicted to have CCd. Since the fungal branch to which yeasts belong is not at the deepest point in the phylogenetic tree (see Figure 1), it is unlikely that these yeasts have primitive types of VAPs. It is reasonable to assume that all ancestors of VAP had CCd from the beginning and that certain yeast lineages lost CCd during evolution.

### 4.7. The Phylogenetic Characteristics of IDR2

The sequence conservation of IDR2 in both VAPA and VAPB is low, and its length varies. In addition, proline and glycine are scattered and have characteristics that inhibit the formation of a stable secondary structure. Thus, they are considered typical IDRs, as predicted by secondary structure prediction programs and the predicted AlphaFold3 structure. However, the C-terminal part of IDR2, the juxtamembrane region, is conserved with concentrated positively and negatively charged amino acids, and VAPB conserves additional acidic residue. The water in the juxtamembrane region is thought to be in a special environment due to the charge on the membrane surface. The sequence of the juxtamembrane region actually affects the interactions of the TMd [90], so the short part of this juxtamembrane region is probably related to its function.

Phosphorylation sites are predicted in both VAPA and VAPB in IDR2, but their number is remarkable for VAPA, whereas the number of phosphorylation sites in IDR1 is remarkable for VAPB, not VAPA. These residues are not completely conserved, but both IDR1 and IDR2 are rich in Ser or Thr residues.

Considering that both VAPA and VAPB have conserved acidic residues in the juxtamembrane region, and that VAPA has additional aspartic acids, if all the reported phosphorylation sites were phosphorylated, the entire IDR would be in a state of considerable negative charge. Such concentrated negative charges may provide the flexible polymer chains the property of preferring a somewhat rigid and extended conformation, which would affect its shrinkage and interactions with other sites and other molecules.

### 4.8. The Phylogenetic Characteristics of the Transmembrane Domain

In 1995, VAP was predicted to have a hydrophobic region at its C-terminus [15,16], and in 2010, experimental evidence demonstrated that this region is necessary for anchoring to the membrane [34]. Although the sequence of this TMd is not as highly conserved as that of MSPd, etc., several common and paralog-specific properties can be found in VAPA and VAPB.

The GxxxG motif is conserved in vertebrate VAPA and VAPB. This motif is considered important for the association of membrane proteins via their TMd [91]. The conserved position of the GxxxG motif is exactly the same in VAPA and VAPB, so if this motif interacts, it is thought to be in the part of the membrane that is furthest from the cytoplasmic side and close to the deepest part of the membrane.

The positive charge of lysine is conserved in exactly the same position closest to the lumen side of the membrane. This seems to restrict the oscillation of the TMd perpendicular to the membrane through electrostatic interaction with the negatively charged hydrophilic lipid head group on the membrane surface, fixing it at the same depth.

A positively charged arginine is also conserved on the cytoplasmic side of the TMd of VAPB; assuming that this also restricts the movement of the TMd, it seems that, for a specific thickness of the lipid bilayer, the helix that constitutes the TMd of VAPB is strongly restricted not only in the depth direction of the membrane but also in the tilt angle. On the other hand, in VAPA, which lacks the R residue on the cytoplasmic side, it is presumed that the restriction on the tilt angle is weaker than that of VAPB.

The presence of amino acids with aromatic rings in TMd is also important for intramembrane association, and more than two phenylalanines are conserved in both VAPA and VAPB (Figure S2). It is thought that the aromatic rings of the intramembrane domains strengthen the TMd–TMd interaction. The location of the phenylalanine clusters is different between VAPA and VAPB.

Considering the aforementioned differences between VAPA and VAPB in terms of R residues, VAPA interacts more intensively closer to the deeper part of the TMd and is less restricted in the shallow part of the membrane, whereas VAPB interacts in various ways between TMds and between TMd and membrane lipids from the deep to the shallow part of the membrane, which may result in differences in the manner of association and the tilt angle of the aggregates. Furthermore, these differences are expected to result in differences between the formation of homodimers or oligomers and the formation of heterodimers or oligomers for VAPA and VAPB, respectively.

## 5. Quantitative Image of Full-Length VAPA/B in Action

### 5.1. Segmental Distribution of IDRs as Unperturbed Flexible Chains

To understand the behavior of VAP on and between the membranes of multiple intracellular organelles, it is essential to understand their overall structures and movements. However, because VAP is a protein in which three domains are connected by the linkers IDR1 and IDR2, which are flexible and have no fixed shape, it is difficult to evaluate its overall structure using only the usual structural biology techniques for proteins with rigid three-dimensional structures. Here, we consider IDR1 and IDR2 as unperturbed flexible polymer chains (completely flexible polymer chains) and consider the distribution of their end-to-end distances. Unperturbed flexible polymer chains generally have a strong tendency to adopt relatively shrunken conformations due to the thermal motion of each segment.

**Figure 8:**
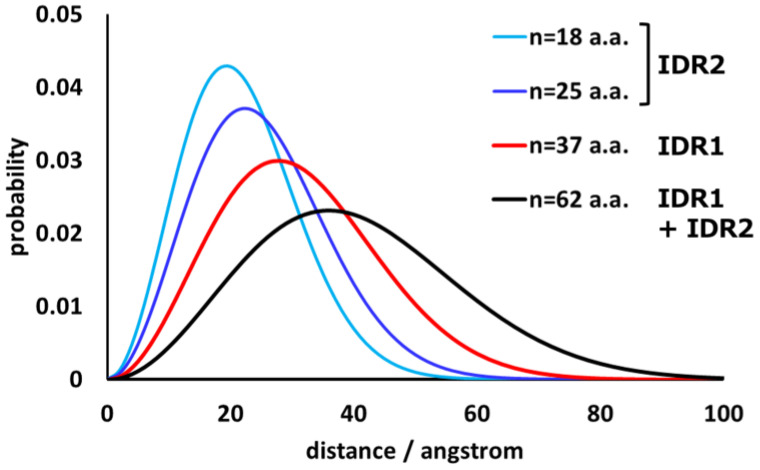
Probability density function of the end-to-end distance of the flexible chains. The number n is the number of amino acid residues in the flexible peptide chains. IDR1 and IDR2 are 37 and 18–25 amino acid residues long, respectively, and the sum of IDR1 and IDR2 was up to 62 amino acid residues long. The conformation changes stochastically according to the distribution of the end-to-end distance.

The shape of the probability density function of the distance between the ends is known [92]. Thus, information about the distance between one end of flexible IDR and the other end can be obtained. Since IDR1 is 37 residues long and IDR2 is 18–25 residues long, the distribution (probability density function) of the distance between each end can be calculated as follows:

(1) 4πr2p0R=4πr2·32πR20exp−3r22·R20

(2) $${\langle R^{2}\rangle }_{0}=C_{\infty }nb^{2},$$

where *r* is the end-to-end distance; 〈*R*^2^〉_0_ is the mean-square end-to-end distance; *C*_∞_ is the limit characteristic ratio, which is a measure of the inherent stiffness of the polymer chains; *n* is the number of segments (peptide chain length); and *b* is the segment length [92]. For peptide chains consisting entirely of L-amino acids, *C*_∞_ is usually in the range of 2–9 [93]. Here, the calculation was performed with *C*_∞_ = 2.16 and *b* = 3.8, where *C*_∞_ = 2.16 is the value for poly(Gly), assuming the most flexible limit [94]. The results are shown in Figure 8.

**Figure 9:**
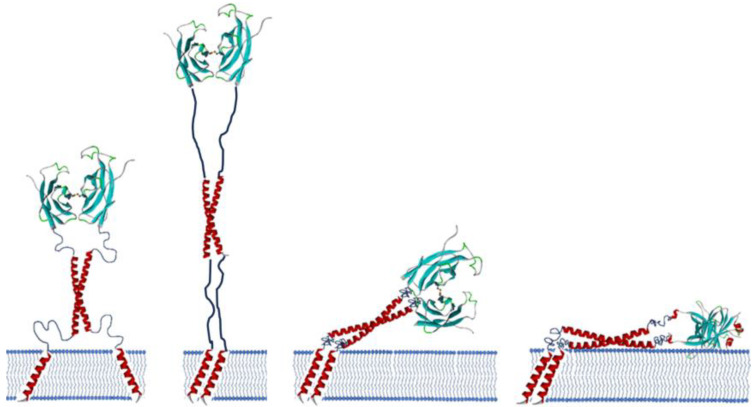
Various predicted structures of VAP, including the state where it is fully extended. Both IDR1 and IDR2 are predicted to move in this manner according to the probability density functions shown in Figure 8.

The polymer chains can adopt extended conformations with low probability under natural thermal motion. The maximum lengths were calculated to be 2.8 × 37 = 103.6 Å (IDR1) and 2.8 × 25 = 70.0 Å (IDR2). The IDR is continuously changing conformation to satisfy the probability density distribution. The CCd of about five heptad repeats (corresponding to a rigid rod of about 55 Å length) is inserted between the two IDRs. When the MSPd of VAP occasionally reaches the farthest position from the transmembrane domain, the vertical distance from the membrane surface to the center of the MSPd is about 230 Å. Therefore, the entire length of VAP is continuously expanding and contracting within a hemisphere of about a 230 Å radius, starting from the transmembrane domain on the membrane. MSPd swings around like a head (Figure 9).

### 5.2. Multivalent Interactions and Local Effective Concentrations

The experimental results confirmed that VAPA and VAPB, when both are full length, can form homodimers, heterodimers, or oligomers [19,26,95]. As mentioned above, crystal structures of MSPd as the homodimer in the apo state or the oligomer with FFAT, have been reported [31,75]. CCd is also a structural motif used for intermolecular interactions, and TMd has a GxxxG motif and aromatic amino acids, which are considered dimer-formation motifs. However, this GxxxG motif is not found in the TMs of many organisms other than vertebrates, and CCd does not appear to exist in yeast. Therefore, the following discussion is limited to vertebrate VAPA/B. Thus, although VAPA/B appears to have the property of multimerization, no clear association is observed with MSPd alone in solutions with concentrations lower than the crystallization concentration [31,65,75]. CCd may also not have a strong affinity for dimerization [19]. However, even if MSPd, CCd, and TMd do not have strong binding strengths on their own, they may well have been designed as effective interactors when used in combination.

This is natural when the following conditions are considered. First, even if the Gibbs energy of the interaction of each domain alone is small enough that it is difficult to observe in biochemical experiments, the association constant under the combined Gibbs energy can be at a level that is easily observable. This is due to a basic property of thermodynamics: even if the interaction energy is additive, the equilibrium constant is a total product [96]. It should be noted that the advantage of multivalent interactions between multiple domains linked by IDRs is not that they are stronger than monovalent interactions between proteins with a normal rigid overall fold. Although weak interactions that are difficult to observe individually can achieve physiologically significant strength in combination, the final binding strength and strength at equilibrium do not reveal the physiological advantage of a multivalent interaction rather than a strong monovalent interaction. On the contrary, in a situation where a flexible portion in such a molecule is fixed by the interaction, it is possible that a trade-off arises, with the loss in terms of energy due to the rapid decrease in entropy caused by the rapid narrowing of the freely selectable conformational space by the binding [93,97].

It is highly likely that the local effective concentration has an effect on the orientation factor. In general, in a collision between molecules in free three-dimensional space, there is no regularity in the relative orientation of both molecules at the time of the collision. Therefore, collisions effective for complex formation are limited to those where the orientation is probabilistically appropriate. On the other hand, if two molecules are anchored by a linker at one end of each molecule and are diffusing in a confined space, their orientation at the time of collision may be initially constrained to a certain extent (Figure 9). This acts on the contents of the orientation factor to increase the probability of effective collisions compared to completely random collisions. However, if the linker is extremely long and flexible, the original orientation is not restricted at all, and this effect is not advantageous. Therefore, the linker portion must have an appropriate length and degree of flexibility in evolution.

Here, we consider the difference between these two types of intermolecular interactions in more detail. Let us now assume that the linker between the domains has no flexibility and consider a rigid protein in which the relative orientation of the interaction surfaces of the three domains is completely fixed. This corresponds to the association between proteins with a normal rigid overall structure. In this case, the energy of the interaction is close to the sum of the energies of multiple weak interactions, resulting in a strong monovalent interaction and equilibrium constant of dissociation/association.

On the other hand, consider a case in which the equilibrium constant of dissociation/association is similar but the linker has some flexibility. The important thing here is that the equilibrium constant is the ratio of the on-rate to the off-rate. When viewed microscopically, the interaction energy of each interaction site alone is not so large, the size of each site is relatively small, the apparent concentration is increased, etc., so it is expected that both the on-rate and the off-rate that make up the apparent equilibrium constant are much larger than those of a rigid molecule. In other words, microscopically, each part is in flux, such that it repeatedly attaches and detaches on a fast time scale. Therefore, even if the monovalent strong interaction and the multivalent interaction consisting of individual weak interactions show the same equilibrium constant for the formation of the aggregate as a whole molecule, the time scale of the microscopic state change is different.

This means that the multivalent type is likely to have the function of switching the shape and interaction partner of each interaction site in response to various changes in the environment in which the molecular group is placed, such as the presence of other molecular groups, the presence or absence of ligand binding, and changes in the dielectric constant, including the presence or absence of nearby membrane components. In this sense, the function achieved by the domain architecture of VAP should be considered not only to statically tether intracellular organelles and keep them at a constant distance but also to be achieved by transitioning between multiple states in a dynamic manner.

As VAP is a membrane protein, its diffusion in cells is two-dimensional rather than three-dimensional. Here, we assume that VAP is mainly present on the ER membrane, estimate its areal density on the ER membrane, and obtain a basis for considering the interaction frequency.

First, we took HeLa cells as an example. Information on the number of VAPA/B molecules per cell is provided in PaxDb [98] for HeLa cells (entry id: 1305695320, *H. sapiens*—Cell line, Hela, SC (Kocher, proteomics, 2014)) as 485 ppm (ENSP00000345656) for VAPA and 46.7 ppm (ENSP00000417175) for VAPB, respectively. The total number of protein molecules in HeLa cells is approximately 1.0 × 10^10^ [99]. Therefore, the number of VAPA and VAPB molecules per cell is 1.0 × 10^10^ × 485 ppm = 4.8 × 10^6^/cell and 1.0 × 10^10^ × 46.7 ppm = 4.7 × 10^5^/cell, respectively.

Next is the area of the ER membrane. The volume of a HeLa cell is about 3000 μm^3^ [99], and if the cell is approximated as a sphere, the total area of the cell plasma membrane is about 1006 μm^2^. The total area of the endoplasmic reticulum membrane is estimated to be about 10–20 times the area of the plasma membrane [100], so it is 10,000–20,000 μm^2^. Therefore, the areal density of VAPA is 2.4–4.8 × 10^2^ molecules/μm^2^. Similarly, the areal density of VAPB is 2.4–4.7 × 10 molecules/μm^2^. If we consider the size of the molecules to be on the 5 nm scale, the VAPA molecules cover approximately 0.6–2% of the total area and approximately 0.06–0.2% for VAPB.

To translate this into a situation where collisions occur according to the laws of mass action in three dimensions, approximating the molecule as a cube with sides of 5 nm, the equivalent concentration *C*_3*D*_ was calculated as follows:

(3) $$C_{3D}=\rho_{2D}\times 2\times 10^{17}=\rho_{2D}\times 2\times 10^{17}/N_{A}\;[mol\cdot L^{-1}]$$

where $N_{A}$ is Avogadro’s number and $\rho_{2D}$ is the number of molecules per 1 μm^2^, which can be obtained from the above areal density.

Thus, the above areal density corresponds to three-dimensional crowding, corresponding to concentrations of approximately 40–120 μM and approximately 4–11 μM, respectively. This is the concentration level at which even weak interactions can reach a certain frequency in equilibrium.

VAPA/B expression is less tissue-specific [101] and is considered ubiquitous by Uniprot. Expression levels of both VAPA and VAPB are reported to be higher in the brain than in other organs [55,56]. Thus, crowding levels in the brain might be expected to be as high, or higher, than in HeLa cells. Note that the amount of VAPA in HeLa cells is about 10 times higher than that of VAPB, whereas the amounts of VAPA and VAPB in the brain are comparable because the expression level of VAPB in the brain is about 10 times higher than in HeLa.

Furthermore, the approximate local effective concentration of each CCd under the condition that two TMd molecules interact even temporarily can be calculated as follows: When IDR2, which is about 20 residues long, freely bends and moves away from the TMD, the average range of motion is within a hemisphere with a radius of about 20 angstroms, so the volume is 1.7 × 10^−26^ m^3^, and the presence of two molecules in this volume corresponds to a local concentration of approximately 200 mM. Of course, under conditions where CCd interactions are established, the local mutual effective concentration of TMd also increases, even if only temporarily, but this movement is limited to within the membrane, and considering that detachment from the membrane is inhibited by basic residues present at the deepest part of TMd, only very narrow two-dimensional movement is allowed, so the local mutual effective concentration ends up being very high.

The local effective concentration of MSPd when CCd interacts can be estimated similarly. In this case, because IR1 has a longer chain length than IR2, the local mutual effective concentration is approximately 60 mM. Thus, it should be considered that the full-length VAP molecule is highly likely to associate and function to a considerable extent in the intracellular environment, even if the strength of the interaction between its individual components is at a low level that is difficult to detect in biochemical experiments. However, we have not considered the collision orientation factor here. Under conditions in which the local mutual effective concentrations of each moving part are high due to dimer or multimer formation, it is likely that favorable conditions also apply for the collision orientation. Thus, in reality, the association will be even more favorable.

## 6. Importance of IDRs

Almost half of the peptide chains in eukaryotes are IDRs [102,103]. Compared to prokaryotic proteins, many eukaryotic proteins are characterized by the presence of multiple structured domains connected by IDRs [104], and IDRs are essential for considering the biological functions of eukaryotes [3]. More than 50% of transmembrane proteins [105], especially on the cytoplasmic side, have IDRs [106] and are involved in many important physiological processes, such as G protein-coupled receptor and cytokine receptor signaling [107,108], cadherin-mediated cell–cell junctions [109], and so on [110]. In neurons, VAPs and potassium channels form clusters through the interaction of the MSPd of VAPs with the FFAT motif of potassium channels located at the C-terminal IDR, and these clusters contribute to neuronal function [111,112,113,114,115]. The presence of IDR in assembler proteins is involved in the formation of large complexes [116]. VAPs could be the assembler proteins that possess IDRs, and the FFAT motifs that interact with VAPs are often found in IDRs. This could lead to the formation of large complexes, and more than 100 proteins share the FFAT motif [4], making the interaction between IDRs and VAPs very important.

## 7. Relationship Between VAP and Disease

As described above, the VAP gene has been conserved since the very early stages of eukaryotic evolution. VAP interacts with a large number of proteins [8] and plays diverse roles. Not surprisingly, the function of VAP is related to the basis of eukaryotic life activities, and indeed, it has been reported that the homozygous null VAPA gene in mice results in embryonic lethality [117], and the VAPs are associated with numerous pathologies [14]. The relationship between VAP and disease can be divided into two groups: (1) the function of VAP is hijacked by pathogenic microorganisms, including various viruses; and (2) the function of VAP is impaired by the mutation and/or the other mechanisms.

Group (1) is the hijacking of VAP by viruses and bacteria. VAP is used for replication by pathogens such as adenovirus, Aichi virus (AiV), chlamydia, hepatitis C virus (HCV), herpes simplex virus-1 (HSV-1), norovirus, poliovirus, and rhinovirus [14]. Some of these interact with VAP through the FFAT motif, while others interact in other ways [14].

VAPA/B recruits OSBP to the ER membrane by binding to the FFAT motif of OSBP [65,118]. Recently, VAP has been reported to be involved in SARS-CoV-2 infection [119] and *Legionella pneumophila* infection [120]. In SARS-CoV-2 infection, SARS-CoV-2 Nsp, Nsp3, Nsp4, and Nsp6, which are involved in the formation of double-membrane vesicles, a key step in viral replication, interact with both VAP and OSBP. The RNA-dependent RNA polymerase of SARS-CoV-2 contains the FFAT-like motif that binds VAP [33]. In Legionella infection, VAP, OSBP, and the phosphatidylinositol 4-phosphate (PtdIns(4)P) phosphatase Sac1 form a Legionella- and host-cell-driven PtdIns(4)P lipid gradient at the Legionella-containing vacuole (LCV)-ER MCS, a unique membrane-bound replication compartment, to promote pathogen vacuole maturation.

For group (2), numerous reports are linking VAP to diseases, cystic fibrosis (CF) [121,122], and neurological disorders, such as Alzheimer’s disease (AD) [40,43,123], Parkinson’s disease (PD) [124], Lewy Body Disease (LBD) [125], frontotemporal dementia (FTD), amyotrophic lateral sclerosis (ALS) [126], and multiple system atrophy [127]. The P56S mutation in VAPB has been the most extensively studied in terms of the relationship between mutations in the VAP gene and ALS. Other known missense mutations associated with ALS include T46I [128], P56H [129], A145V [130], and V234I [131]. In addition, a deletion associated with ALS has been reported for S160Δ [132]. Detailed reviews of disease-associated mutations can be found in [12,14]. Of these, a V234I patient has C9orf72 repeat expansions, and S160Δ has been detected in both patients and controls [131].

In addition, many missense mutations are reported in ClinVar [133]. The mutation numbers are 1 in the N-terminal region, 3 in MSPd, 20 in IDR1, 3 in CCd, 1 in IDR2, and 0 in TMd for VAPA; and 4 in the N-terminal region, 55 in MSPd, 15 in IDR1, 11 in CCd, 10 in IDR2, and 16 in TMd for VAPB. Note that the residue numbers in ClinVar are defined as the numbers corresponding to the longer splicing variant (with longer IDR1), which is not canonical in UniProt [134]. The effects of many of these mutations are still unknown.

Future efforts to distinguish mutations that affect function from those that do not will be helpful in understanding the mechanisms of functional expression of VAPs. In general, finding a correlation between disease and the presence of mutations or symptoms (pathology) and the causes of disease (pathogenesis) has been a challenge for more than a century [135]. Accurately linking mutations to disease remains challenging even with recent advances in technologies such as genome-wide association studies (GWAS), machine learning, and gene editing [136]. To fully understand these mutations of VAPs in pathology and pathogenesis, it will be necessary to gain an interdisciplinary understanding of diseases, e.g., through protein science research on VAPs and their interacting proteins, as well as research at different levels, such as cell biology, histopathology, and clinical manifestations.

## 8. Perspectives

As mentioned above, the full length of VAP without any interactions with other molecules is expected to move in a hemisphere of about 230 Å radius, starting from the transmembrane site on the membrane and swinging MSPd around like a head while expanding and contracting and searching for a binding partner. The frequency of realizing a conformation close to this maximum perpendicular distance during thermal motion is not very high, judging from the shape of the probability density function. The average intermembrane distance at MCSs is 6–15 nm between ER and mitochondria [130], 3–15 nm between ER and endosomes [137], and 19–22 nm between ER and the plasma membrane [138]. Considering the proteins possessing FFAT motifs on the opposite membrane are of a certain size, this movement of VAP is intriguing because it indicates that tethering by VAP and FFAT can occur with a certain probability even between membranes that are much farther apart than the average gap at the MCS. Furthermore, as is clear from the probability density function in Figure 8, both IDR1 and IDR2 are likely to shrink to the distance region with the highest probability density—i.e., the region near 30 Å and 20 Å, respectively—immediately after adopting this extended conformation. Therefore, if the extended VAP successfully captures FFAT stochastically, it is expected that it will pull it towards the membrane where it is anchored. This idea is supported by the recent publication showing the significance of IDR [3,139].

This property resembles the formation of a coiled-coil zipper and the associated power stroke resulting from the coiled-coil domains of SNARE proteins during membrane fusion. Because the CCd of VAP is originally similar to the CCd of SNARE proteins, it is not surprising that it has the ability to form a coiled-coil zipper-like complex through interactions with other factors. Therefore, it is conceivable that the result of thermal motion is the sequential formation of new complexes. In this case, it should be noted that these are not energy-consuming processes that require coupled reactions such as ATP hydrolysis but, rather, changes resulting from the thermal motion of each segment, reminiscent of a Brownian ratchet mechanism.

As we have seen, the molecular characteristics that VAP has maintained over hundreds of millions of years of evolution seem to support highly dynamic function expression due to the flexibility of the entire molecule and the multivalent and rapid exchange properties of the interactions. These dynamic characteristics of VAP may facilitate the function of the lipid transport [14]. For example, at the interface between the ER and other organelles, the VAP-interacting protein CERT is involved in ceramide transport [21,140,141,142], and OSBP [143] and STARD3 [32,144] are involved in cholesterol transport.

**Figure 10:**
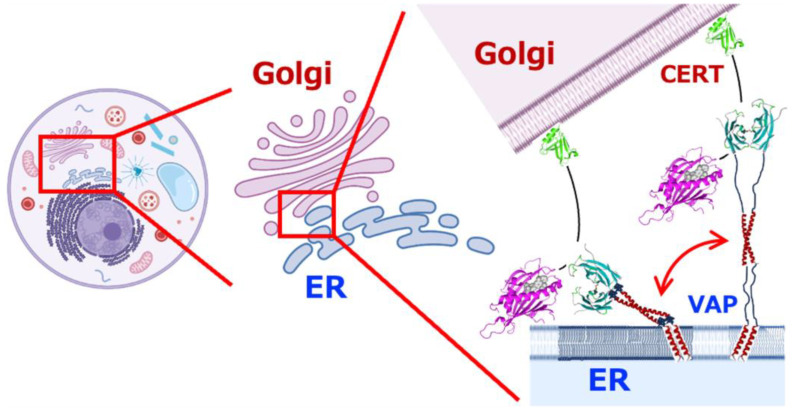
A schematic diagram showing how VAPs tether and attract two intracellular organelles via the Brownian ratchet mechanism. The diagram shows how VAPs on the ER membrane interact with ceramide transfer protein (CERT) [145], which is anchored to the membrane of the Golgi apparatus, via the FFAT motif of CERT. This demonstrates the dynamic process achieved by multivalent interactions between multiple domains connected by two IDRs, which is important for their function. The PH and START domains of CERT are green and magenta, respectively, and the unknown structural part connecting them is shown with a black line. The FFAT motif of CERT is located on the black line near the START domain.

If we interpret the meaning of the characteristic architecture of VAP from this perspective, it is possible that the various target proteins and cooperatively functioning proteins that have been reported to interact with VAP may have similar properties. The structures of various proteins involved in MCS or tethering of intracellular organelles are predicted to have flexible IDRs in many cases. This strongly suggests that the interaction between intracellular organelles is not a static type of interaction between rigid proteins with high selectivity and a slow exchange rate between on–off states but a system based on dynamic and stochastic control, such as the Brownian ratchet mechanism examined here (Figure 10). This area requires further research.

## Figures and Tables

**Table 1:** Experimentally determined structures of MSPd (PDB).

PDB ID	Molecule	Species	MSPd Chain	FFAT Chain	Method	Resolution	Comments
6lp4	Scs2p	yeast	A		X-ray	2.049 Å	
3ikk	VAP-B	human	A-B		X-ray	2.5 Å	S-S dimer
2mdk	VAP-B	human	A		NMR		P56S in DPC
7x14	VAP-B	mouse	A	B	X-ray	1.675 Å	
2cri	VAP-A	mouse	A		NMR		
1z9l	VAP-A	rat	A		X-ray	1.7 Å	
1z9o	VAP-A	rat	A, B, C, D, E, F	G, H, I, J, K, L	X-ray	1.9 Å	include non S-S
2rr3	VAP-A	human	A	B	NMR		
6tqr	VAP-A	human	A, B, C, D	E, F	X-ray	1.85 Å	S-S dimer
6tqs	MOSPD2	human	A, B, C, D, E, F	G, H, I, J, K	X-ray	2.25 Å	
6tqt	MOSPD2	human	A		X-ray	1.5 Å	
6tqu	MOSPD2	human	A, B	C, D	X-ray	2.4 Å	one Cys
1wic	MOSPD2	mouse	A		NMR		

## Data Availability

The datasets and materials used and/or analyzed during the current study are available from the corresponding authors upon reasonable request.

## Supplementary Materials

The following supporting information can be downloaded at: https://www.mdpi.com/article/10.3390/molecules30061220/s1. Figure S1: Multiple sequence alignment of VAPs for eukaryotes; Figure S2: Multiple sequence alignment and phylogenetic tree of VAPs for vertebrates; Figure S3: Evolutionary conservation of individual amino acids in vertebrate VAPA; Figure S4: Sequence logo showing the degree of amino acid conservation in vertebrate VAPA; Figure S5: Evolutionary conservation of individual amino acids in vertebrate VAPB; Figure S6: Sequence logo showing the degree of amino acid conservation in vertebrate VAPB; Figure S7: Evolutionary conservation, side chain exposure, and FFAT contact of VAPA MSPd; Figure S8: Predicted structure of the yeast VAP homolog Scs2p using AlphaFold3; Figure S9: Homology modeling of VAPB CCd dimer, trimer, and tetramer structures; Figure S10: Illustration of local concentration effects of VAPs due to multivalent interactions. References [63,64,146,147,148,149] are cited in the supplementary materials.

## Abbreviations

The following abbreviations are used in this manuscript:

AD: Alzheimer’s disease; ALS: amyotrophic lateral sclerosis; CCd: coiled-coil domain; CERT: ceramide transfer protein; CF: cystic fibrosis; FFAT: two phenylalanines in an acidic tract; FTD: frontotemporal dementia; IDR: intrinsically disordered region; GWAS: genome-wide association studies; LBD: Lewy Body Disease; LLPS: liquid–liquid phase separation; MCS: membrane contact site; MSA: multiple sequence alignment; MSPd: major sperm protein domain; NMR: nuclear magnetic resonance; OSBP: oxysterol-binding protein; PD: Parkinson’s disease; PDB: Protein Data Bank; pLDDT: predicted local distance difference test (a measure of local confidence per residue); PtdIns(4)P: phosphatidylinositol 4-phosphate; PTM: post-translational modification; TMd: transmembrane domain; VAMP: vesicle-associated membrane protein; VAP: VAMP-associated protein; VAPA: VAP-A; VAPB: VAP-B; VAPA/B: VAP-A or VAP-B; WGD: whole-genome duplication; 1R: first round of whole-genome 789 duplication; 2R: second round of whole-genome duplication.
