# Supplementary material for: Beyond Static Tethering at Membrane Contact Sites: Structural Dynamics and Functional Implications of VAP Proteins
Source: Molecules. 2025 Mar 8;30(6):1220. doi: 10.3390/molecules30061220 (PMC11944328; doi:10.3390/molecules30061220)
Supplement: Supplementary file 1 [file molecules-30-01220-s001.zip › molecules-3431503-supplementary.pdf]

Supplementary Materials for

Beyond Static Tethering at Membrane Contact Sites: Structural Dynamics and Functional Implications of VAP Proteins

Takashi S. Kodama[1,*], Kyoko Furuita[1], and Chojiro Kojima[1,2,*]

[1] Institute for Protein Research, Osaka University, 3-2 Yamadaoka, Suita, Osaka 565-0871, Japan.

[2] Graduate School of Engineering Science, Yokohama National University, Tokiwadai 79-5, Hodogaya-ku, Yokohama 240-8501, Japan.

Figure S1: Multiple sequence alignment of VAPs for eukaryotes
Figure S2: Multiple sequence alignment and phylogenetic tree of VAPs for vertebrates
Figure S3: Evolutionary conservation of individual amino acids in vertebrate VAPA
Figure S4: Sequence logo showing the degree of amino acid conservation in vertebrate VAPA
Figure S5: Evolutionary conservation of individual amino acids in vertebrate VAPB
Figure S6: Sequence logo showing the degree of amino acid conservation in vertebrate VAPB
Figure S7: Evolutionary conservation, side chain exposure, and FFAT contact of VAPA MSPd
Figure S8: Predicted structure of the yeast VAP homolog Scs2p using AlphaFold3
Figure S9: Homology modeling of VAPB CCd dimer, trimer, and tetramer structures
Figure S10: Illustration of local concentration effects of VAPs due to multivalent interactions

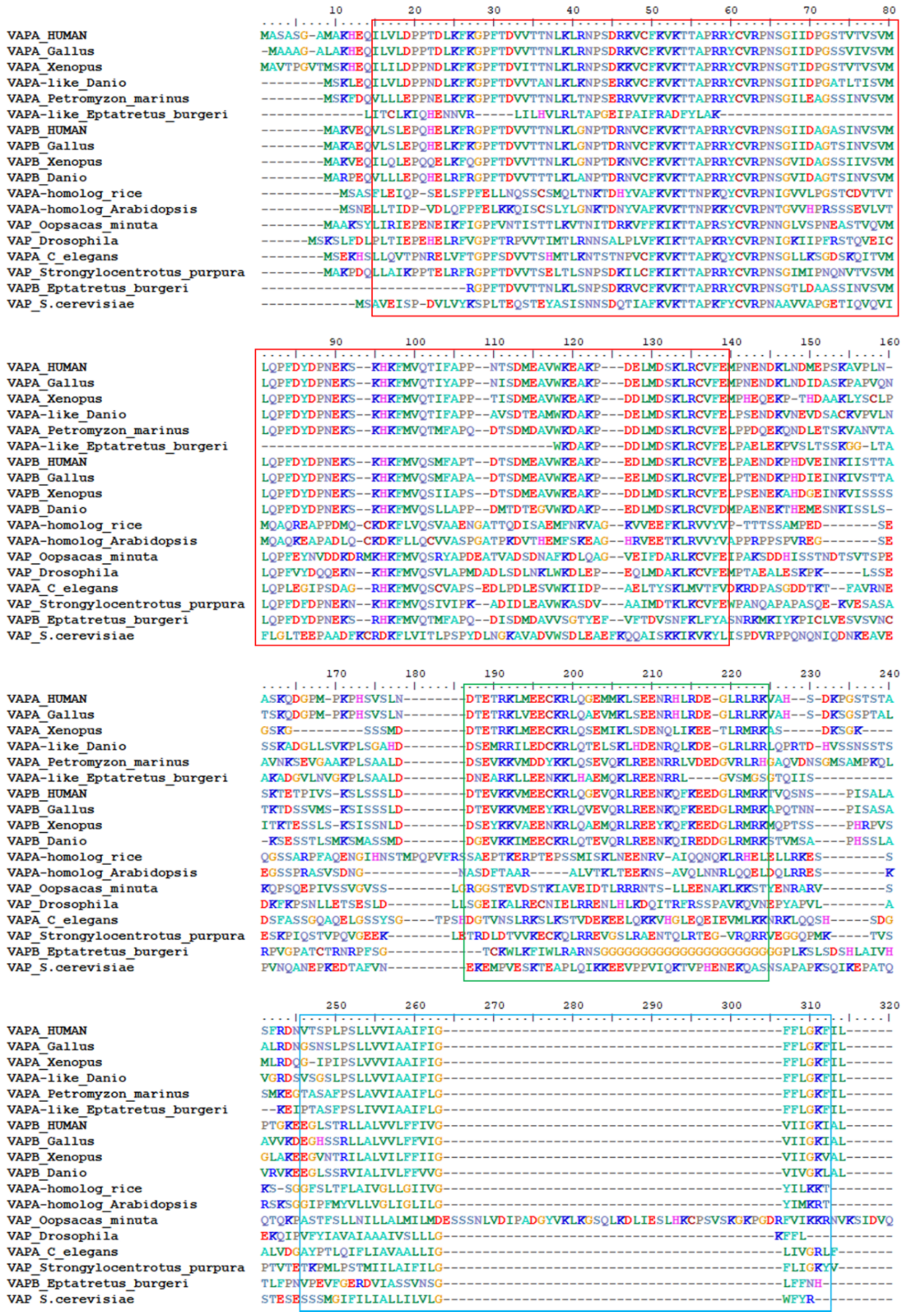

Figure S1. Multiple sequence alignment (MSA) of VAPs for eukaryotes. The regions shown in red, green, and light blue boxes represent MSPd, CCd, and TMd. CCd and TMd are not clearly defined for some eukaryotes.

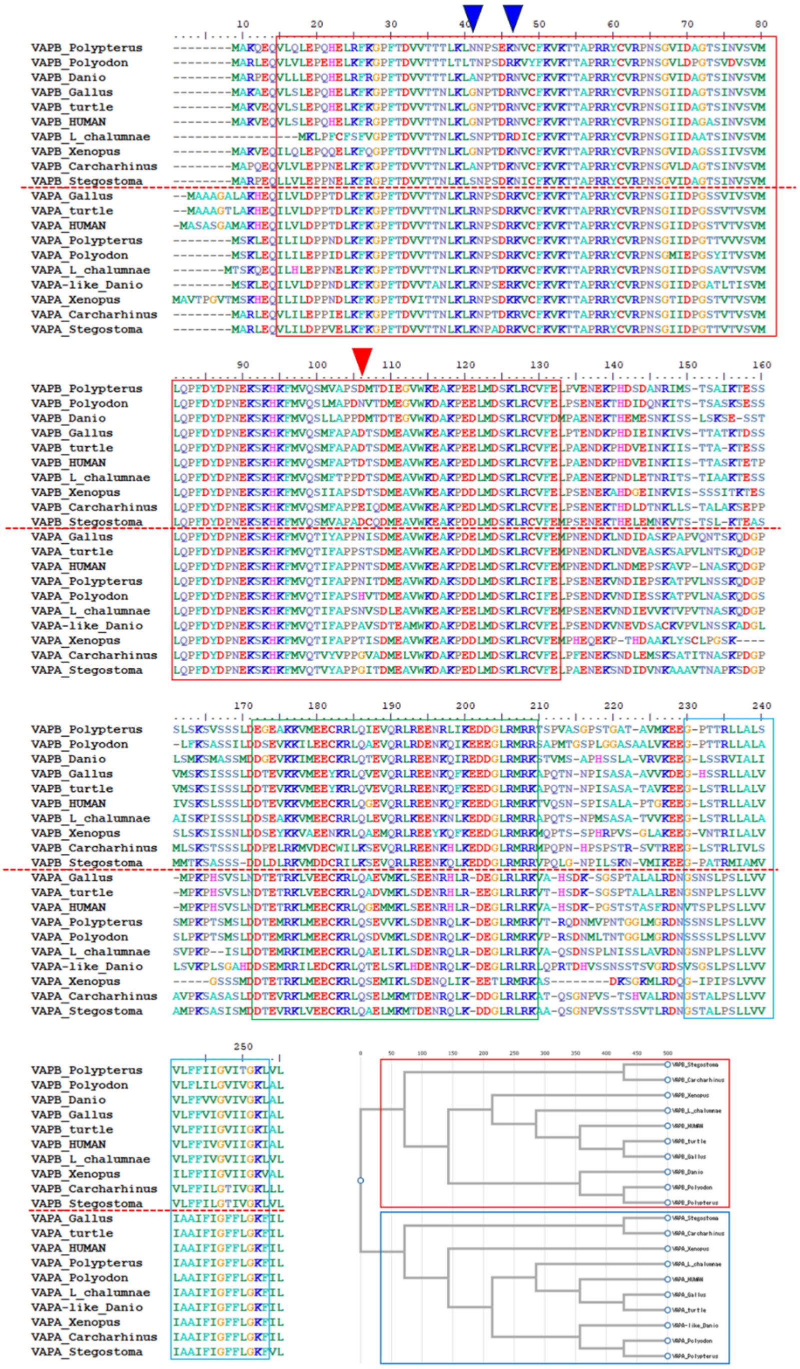

Figure S2. Same as Figure S1 but for vertebrates. The regions shown in red, green, and light blue boxes represent MSPd, CCd, and TMd, respectively, as in Figure S1. The blue triangles indicate the positions of two basic residues characteristically conserved in VAPA, and the red triangle indicates the position of one acidic residue characteristically conserved in VAPB. In TMd, more than two phenylalanines are conserved in both VAPA and VAPB, but at different positions. The phenylalanines in TMd strengthen the TMd-TMd interaction [1,2]. (Bottom right) Phylogenetic tree of VAP proteins. The VAPA and VAPB orthologues each form a monophyletic branch.

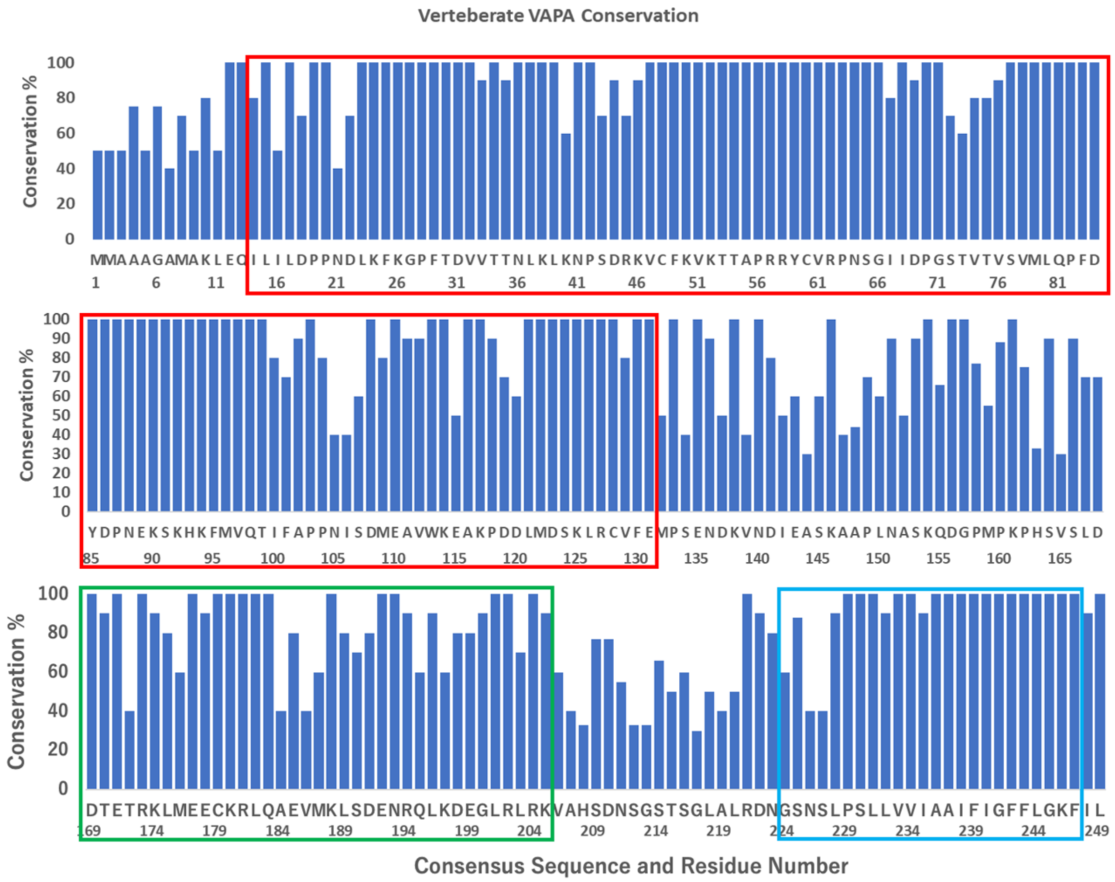

Figure S3. Evolutionary conservation of individual amino acids in vertebrate VAPA. The residue numbers shown at the bottom are those in the human sequence. The degree of conservation of each amino acid in VAPA was calculated using the Consurf server [3] by providing the vertebrate MSA (Figure S2). The method of calculation for evolutionary conservation was set to Bayesian. The regions shown in red, green, and light blue boxes represent MSPd, CCd, and TMd, respectively, as in Figure S1.

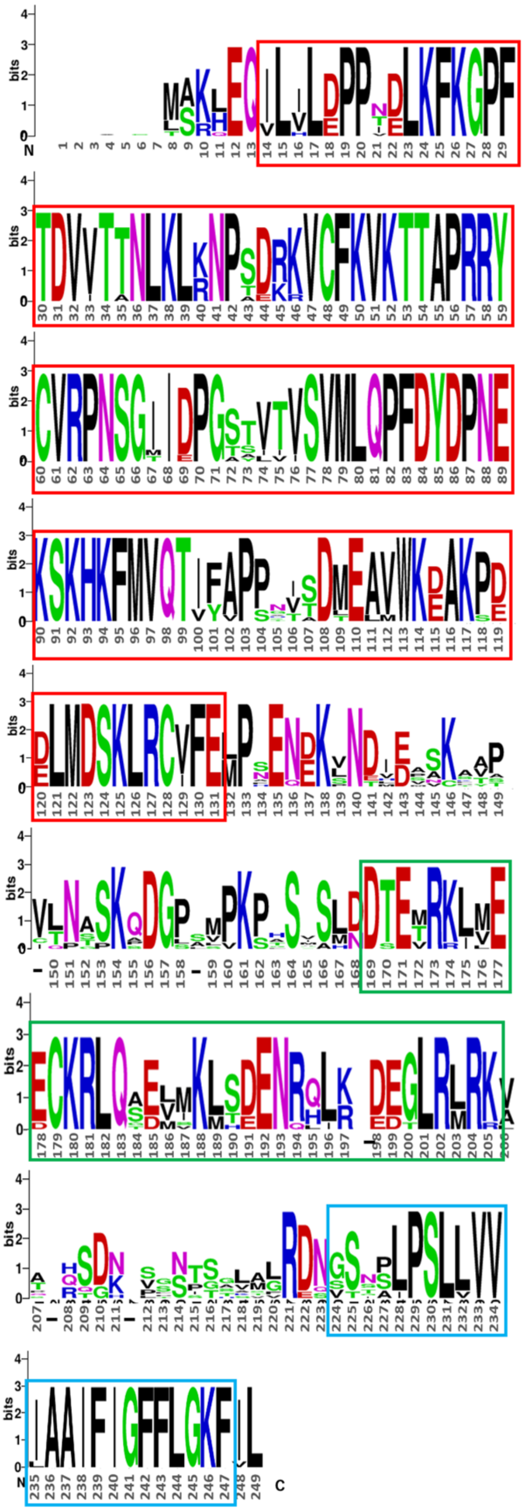

Figure S4. Sequence logo showing the degree of amino acid conservation in vertebrate VAPA. The sequence logo has spaces to allow direct comparison of VAPA and VAPB. The residue numbers below the logo represent the residue numbers in the human sequence. The logo was created by WebLogo [4] using the MSA (Figure S2). The regions shown in red, green, and light blue boxes represent MSPd, CCd, and TMd, respectively, as in Figure S1.

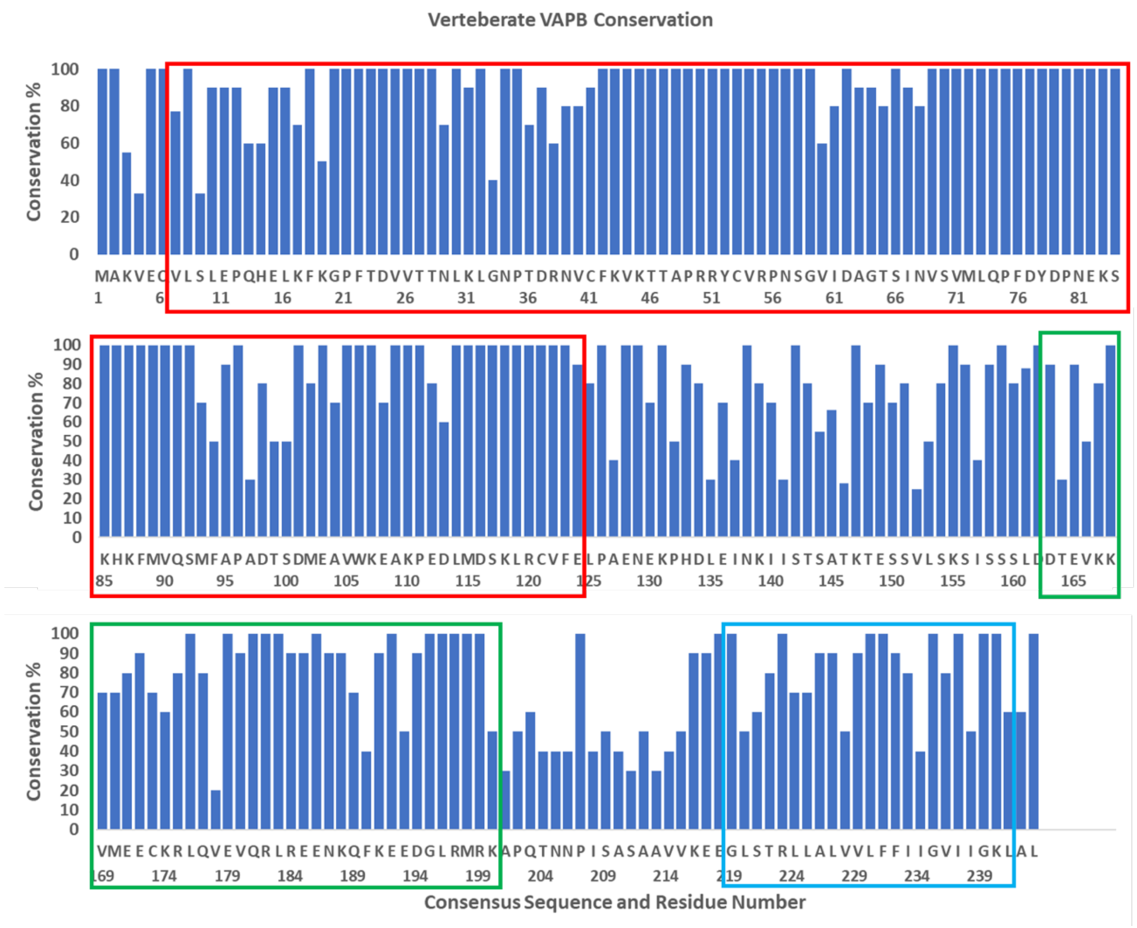

Figure S5. Same as Figure S3 but for vertebrate VAPB.

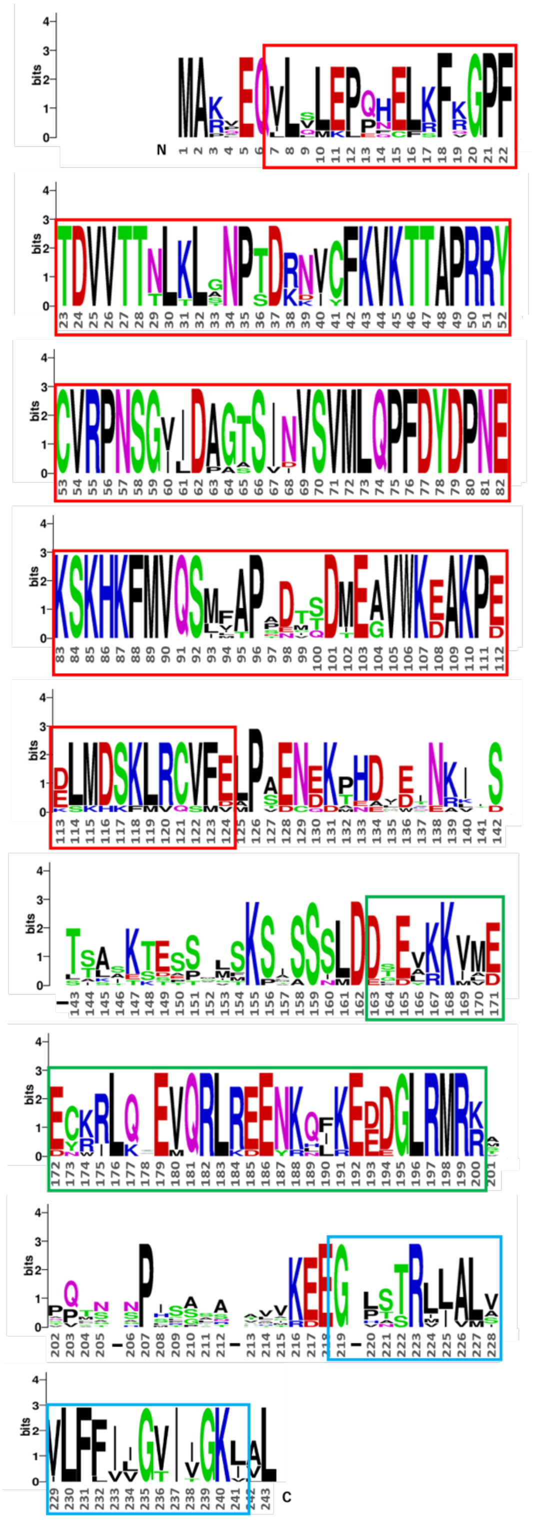

Figure S6. Same as Figure S4 but for vertebrate VAPB.

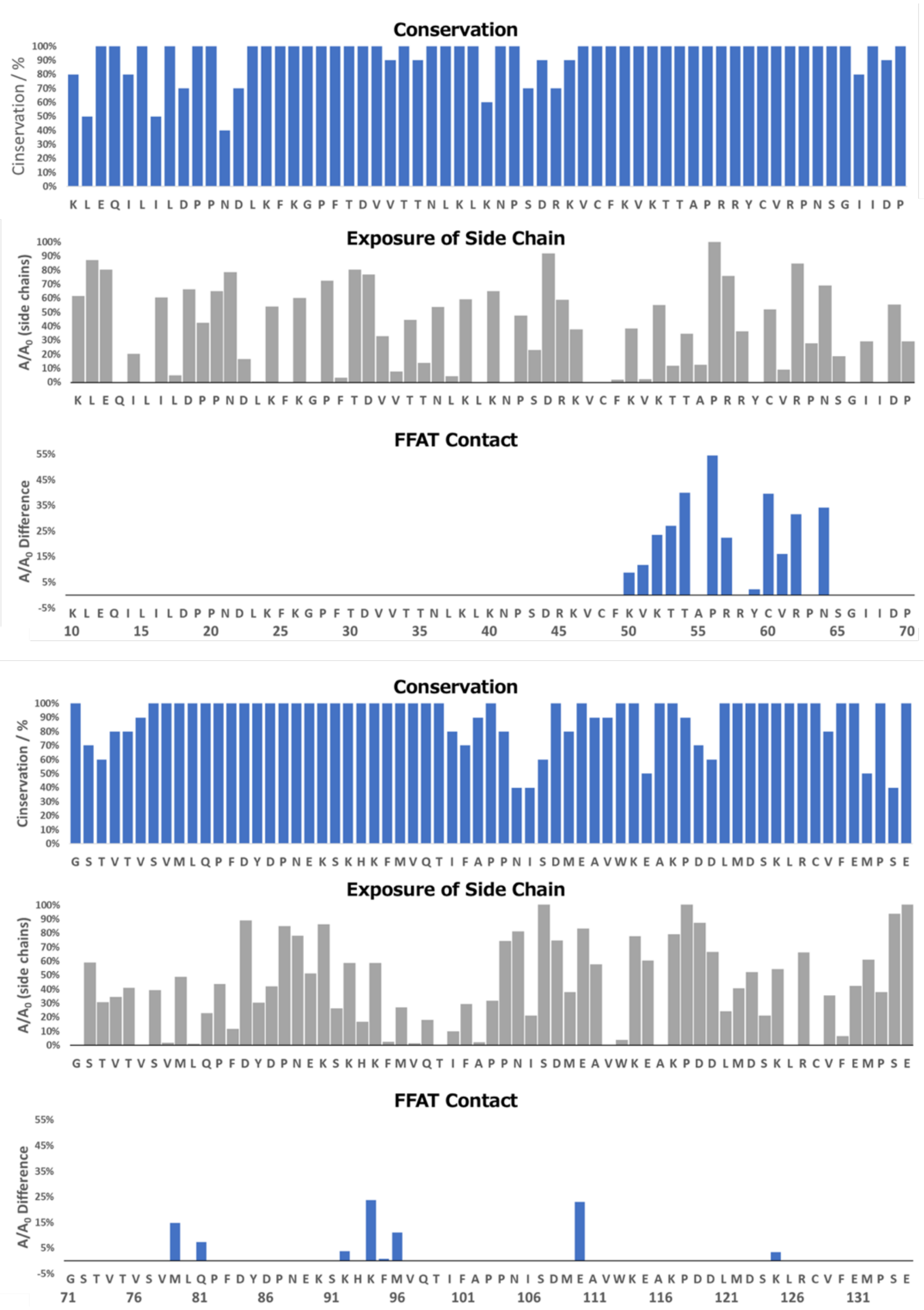

Figure S7. Evolutionary conservation, side chain exposure, and FFAT contact of VAPA MSPd. A_0 is the Solvent Accessible Surface Area (SASA) of each side chain when fully exposed to the solvent, and A is the SASA in the actual three-dimensional structure. A_0 is calculated as the solvation area when the amino acid X is in the middle of the pentapeptide GGXGG, which has no specific secondary structure. Side chain exposure for the VAPA structure (PDB ID: 2RR3) was calculated using STRIDE [5] modified to report main chain and side chain results separately. The degree of FFAT contact was calculated as the difference in A/A_0 between the apo state and the complex state of OSBP with the peptide containing the FFAT motif. This value is 100% when a given residue is completely exposed in the apo state and completely buried in the complex state.

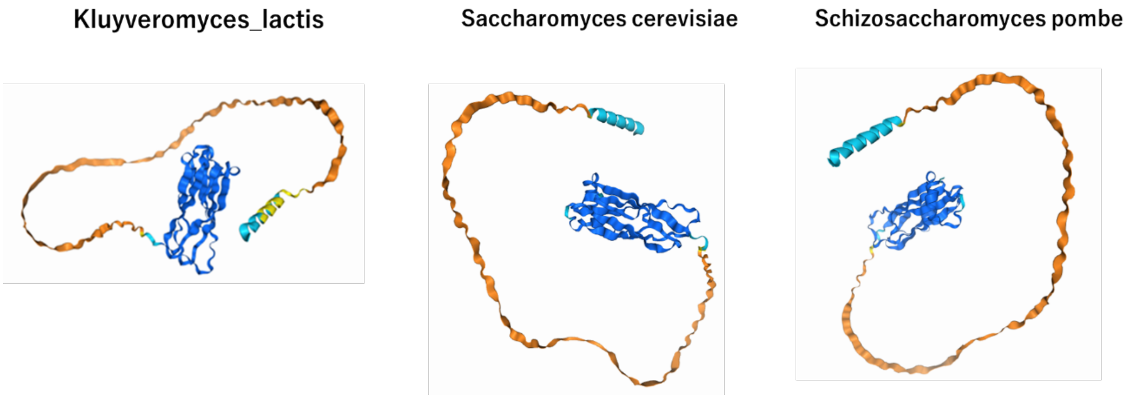

Figure S8. Predicted structure of the yeast VAP homolog Scs2p using AlphaFold3. The region corresponding to CCd in vertebrate VAP proteins is predicted to be part of the long IDR region with low pLDDT values.

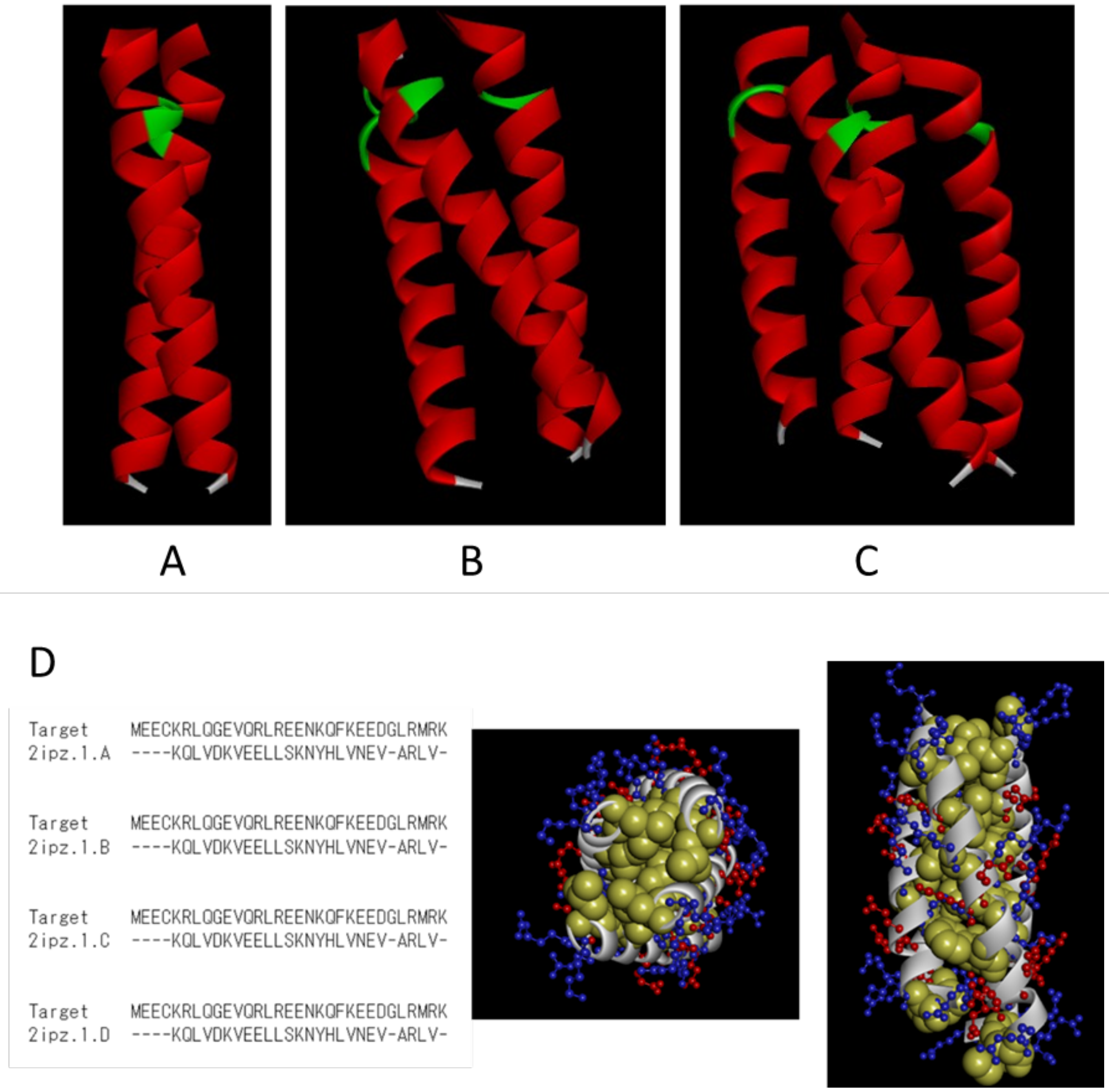

Figure S9. Homology modeling of VAPB CCd dimer (A), trimer (B), and tetramer (C, D) structures. The CCd sequence of VAP has typical heptad repeat characteristics of a general coiled-coil structure. Homology modeling is performed under good conditions using SWISS MODEL [6] based on several crystal structures of GCN4. The templates are PDBID: 2ahp, 2o7h, and 2ipz, respectively. (D) The molecules can associate through the heptad repeat interface and the amphipathic helix is stabilized by electrostatic interactions.

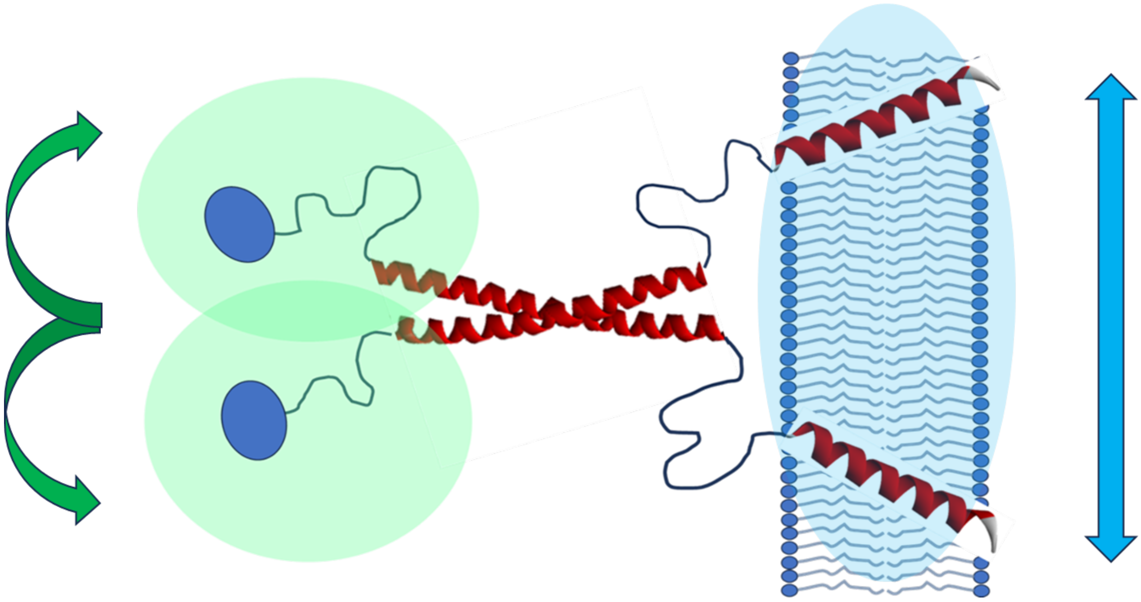

Figure S10. Illustration of local concentration effects of VAPs due to multivalent interactions. The light green and light blue circles and arrows indicate the ranges within which MSPd and TMd can diffuse under conditions in which CCd dimerizes.

References to Supplementary Materials

1. Unterreitmeier, S.; Fuchs, A.; Schäffler, T., Heym, R.G.; Frishman, D.; Langosch, D. Phenylalanine promotes interaction of transmembrane domains via GxxxG motifs. *J Mol Biol.* **2007**, 374, 705–718, doi: 10.1016/j.jmb.2007.09.056.

2. Teese, M.G.; Langosch, D. Role of GxxxG Motifs in Transmembrane Domain Interactions. *Biochemistry* **2015**, 54, 5125–5135, doi: 10.1021/acs.biochem.5b00495.

3. Yariv, B.; Yariv, E.; Kessel, A.; Masrati, G.; Chorin, A. Ben; Martz, E.; Mayrose, I.; Pupko, T.; Ben-Tal, N. Using evolutionary data to make sense of macromolecules with a "face-lifted" ConSurf. *Protein Sci.* **2023**, *32*, doi:10.1002/PRO.4582.

4. Crooks, G.E.; Hon, G.; Chandonia, J.M.; Brenner, S.E. WebLogo: a sequence logo generator. *Genome Res.* **2004**, *14*, 1188–1190, doi:10.1101/GR.849004.

5. Frishman, D.; Argos, P. Knowledge-based protein secondary structure assignment. *Proteins Struct. Funct. Bioinforma.* **1995**, *23*, 566–579, doi:10.1002/prot.340230412.

6. Waterhouse, A.; Bertoni, M.; Bienert, S.; Studer, G.; Tauriello, G.; Gumienny, R.; Heer, F.T.; de Beer, T.A.P.; Rempfer, C.; Bordoli, L.; Lepore, R.; Schwede, T. SWISS-MODEL: homology modelling of protein structures and complexes. *Nucleic Acids Res.* **2018**, 46, W296–W303, doi:10.1093/nar/gky427.
